# Multi-Objective Optimization and Prediction of Mechanical Properties of Green Basalt Fiber-Reinforced Concrete Using Evolutionary ML Algorithms

**DOI:** 10.3390/ma19163436

**Published:** 2026-08-13

**Authors:** Abdullah Al Mamun, Manal Aburizaiza, Wadea Sindi, Muhammad Imran Khan, Md Ehtesamul Haque, Ammar Al-Shayeb, Ziad Shatnawi, Md Kamrul Islam, Muhammad Ali Martuza, Md Arifuzzaman

**Affiliations:** 1 Lyles School of Civil and Construction Engineering, Purdue University, West Lafayette, IN 47907, USA; 2 Department of Computer Science and Engineering, Yanbu Industrial College, Yanbu Industrial City 46454, Saudi Arabia; 3 Jeddah Development Authority, Jeddah 46451, Saudi Arabia; 4 Civil Engineering Department, College of Engineering, Imam Mohammad Ibn Saud Islamic University (IMSIU), Riyadh 11432, Saudi Arabia; 5 Department of Computer Science, The College of Computer Sciences & Information Technology (CCSIT), King Faisal University, P.O. Box 380, Al-Ahsa 31982, Saudi Arabia; 6 Department of Civil and Environmental Engineering, College of Engineering, King Faisal University, P.O. Box 380, Al-Ahsa 31982, Saudi Arabia; 7 Department of Computer Engineering, College of Computer, Qassim University, Buraydah 51452, Saudi Arabia

**Keywords:** basalt fiber-reinforced concrete, evolutionary machine learning, multi-objective optimization, explainable artificial intelligence, sustainable concrete mix design

## Abstract

Basalt fiber-reinforced concrete (BFRC), reinforced with chopped basalt fibers having lengths ranging from 12 to 30 mm and diameters ranging from 0.013 to 0.020 mm, is a sustainable construction material with enhanced strength and durability; however, its complex nonlinear behavior makes accurate prediction and optimal mix design challenging. This study proposes an integrated machine learning framework combining evolutionary optimization, multi-objective optimization, and explainable artificial intelligence (XAI) for BFRC strength prediction and mix design optimization. The proposed framework further incorporates a graphical user interface (GUI) deployment to enhance practical usability and support engineering decision-making. Random Forest, Gradient Boosting Regressor, and XGBoost models were optimized using Genetic Algorithms, Particle Swarm Optimization, and Differential Evolution, while NSGA-II was employed to identify optimal trade-offs between compressive strength and splitting tensile strength. SHAP analysis was applied to interpret the influence of key mix parameters on strength prediction. The optimized models achieved high prediction accuracy, with R^2^ values of 0.88 for compressive strength and 0.95 for splitting tensile strength, demonstrating the effectiveness of the proposed framework. The developed GUI provides a practical decision-support tool for sustainable and performance-oriented BFRC mix design.

## 1. Introduction

Basalt fiber-reinforced concrete (BFRC) has emerged as a promising advanced construction material due to its high mechanical performance, corrosion resistance, environmental friendliness, and cost-effectiveness compared to traditional steel and synthetic fiber-reinforced concretes [1]. Recent studies have demonstrated that sustainable concrete materials can significantly reduce environmental impacts while maintaining satisfactory structural performance [2,3]. In this context, BFRC represents a green construction approach by improving mechanical performance, enhancing durability, and promoting efficient material utilization. In addition, eco-friendly cementitious composites reinforced with innovative fibers have shown improved strength, durability, and long-term serviceability [4,5]. Furthermore, basalt fiber-reinforced composite materials have exhibited enhanced mechanical properties due to improved fiber–matrix interfacial bonding and crack-bridging mechanisms [6]. Basalt fibers enhance crack resistance, tensile strength, and post-cracking performance, making BFRC highly suitable for structural and infrastructure projects in severe environmental conditions [7]. Recent investigations on advanced cement-based composites have further demonstrated that fiber-reinforced materials exhibit improved tensile behavior and long-term mechanical stability under different loading conditions [8]. However, the mechanical behavior of BFRC is strongly influenced by various mix design parameters, including cement content, aggregate proportions, water–cement ratio, fiber dosage, fiber geometry, admixture content, and curing conditions [9]. These parameters exhibit complex, coupled, nonlinear interactions, making the accurate prediction and optimal design of BFRC mix proportions exceptionally challenging [10].

The mechanical properties of BFRC are evaluated through standardized concrete testing procedures. The compressive strength of concrete specimens is commonly determined using the cylinder compression test according to ASTM C39/C39M [11], which specifies the procedures for specimen preparation, loading conditions, and strength calculation. Similarly, the splitting tensile strength is evaluated using the indirect tensile test method described in ASTM C496/C496M [12]. These standardized testing methods provide reliable and consistent measurements of concrete strength properties and are widely applied for conventional and fiber-reinforced concrete evaluation.

Consequently, traditional experimental and empirical approaches rely heavily on extensive laboratory testing and trial-and-error procedures [13]. These methods are time-consuming and expensive, and they frequently fail to address the intricate nonlinear interactions among the mix constituents and fiber properties [14]. While statistical regression models have been employed to predict material strength, their inability to capture highly nonlinear behaviors and multivariate interactions limits their efficacy in complex fiber-reinforced concrete systems [15].

Recent advances in artificial intelligence (AI) and data-driven modeling have significantly improved the prediction of concrete mechanical properties, reducing the reliance on extensive experimental testing [16,17]. Machine learning (ML) algorithms integrated with optimization techniques have demonstrated a strong capability in modeling complex nonlinear relationships among material parameters in civil engineering applications [18,19]. These developments have enhanced prediction accuracy and provided efficient decision-support tools for engineering design and material optimization [20].

Over the past several years, ML methods have demonstrated strong potential in mapping nonlinear material behaviors directly from experimental datasets. Ensemble learning algorithms—such as Random Forest (RF), Gradient Boosting Trees (GBT), and eXtreme Gradient Boosting (XGBoost)—have demonstrated a strong capability in capturing complex nonlinear relationships among material parameters [21]. Though highly effective, the predictive performance of these ML models relies heavily on selecting suitable hyperparameters [22]. Conventional methods, such as gradient-based tuning or grid search optimization algorithms, are computationally expensive and prone to converging on suboptimal solutions, particularly within high-dimensional hyperparameter spaces [23].

Conversely, evolutionary optimization methods, such as Genetic Algorithms (GAs), Particle Swarm Optimization (PSO), and Differential Evolution (DE), possess powerful global search capabilities. They can automatically establish optimal hyperparameter configurations while systematically avoiding premature convergence to local optima [24]. Integrating evolutionary algorithms with ML models can significantly enhance prediction accuracy, robustness, and generalization when handling heterogeneous experimental data such as those of BFRC [25].

Although the majority of existing literature focuses primarily on strength prediction, practical engineering design demands more than just predictive accuracy; mix proportions must be systematically optimized to satisfy multiple, often conflicting objectives [26]. In BFRC mix design, compressive strength and splitting tensile strength must be optimized simultaneously within practical material constraints [27]. Multi-objective optimization frameworks, particularly the Non-dominated Sorting Genetic Algorithm II (NSGA-II), provide an efficient approach to exploring the trade-offs between these competing objectives, ultimately generating a Pareto-optimal front [28].

Furthermore, the ‘black-box’ nature of advanced ML models often restricts their widespread adoption in civil engineering practice. This limitation can be effectively overcome using explainable artificial intelligence (XAI) methods, such as SHapley Additive exPlanations (SHAP), which offer transparent insights into feature importance and nonlinear interactions [29]. Finally, bridging the gap between scientific research and field application requires user-friendly tools, thereby encouraging the development of graphical user interfaces (GUIs) to deploy these sophisticated AI-driven models [30,31]. Recent research has increasingly focused on developing sustainable and low-carbon cementitious materials to reduce the environmental impact of construction while maintaining structural performance [32].

To facilitate precise prediction, optimal design, and practical implementation in BFRC design, this study proposes a comprehensive, integrated framework. The framework combines evolutionary-optimized machine learning models, multi-objective optimization, explainable artificial intelligence, and a deployable GUI. In addition to improving mechanical performance, the proposed framework supports green concrete development by enabling efficient material utilization and reducing unnecessary cement consumption through optimized BFRC mix design. By identifying high-performance mixtures with balanced strength and material efficiency, the framework contributes to sustainable construction practices.

This study focuses on compressive strength (CS) and splitting tensile strength (STS) because these are the most consistently reported mechanical properties in publicly available BFRC datasets and are widely recognized as primary indicators of concrete mechanical performance. Although shear strength is also an important parameter for evaluating the load-bearing capacity of BFRC structural elements, sufficient standardized experimental data were not available to develop and validate reliable machine learning models. Therefore, shear strength prediction is beyond the scope of the present study.

### 1.1. Research Contributions

The main contributions of this study are as follows

- Developing an ensemble ML-based predictive model that combines Random Forest, Gradient Boosting Regressor, and Extreme Gradient Boosting models to precisely determine the compressive and splitting tensile strengths of concrete reinforced with basalt fibers.
- Employing a multi-objective optimization model based on the NSGA-II algorithm to compute Pareto-optimal BFRC mix ratios, enabling the simultaneous optimization of compressive and splitting tensile strengths. Performing a systematic improvement of predictive accuracy and robustness of ML models using heterogeneous BFRC datasets and a combination of evolutionary hyperparameter optimization algorithms (GA, PSO, and DE).
- Identifying the predominant BFRC mix parameters and providing actionable engineering insights into material behavior using explainable artificial intelligence (SHAP analysis) to interpret optimized machine learning models. Designing an independent GUI to facilitate real-time prediction, Pareto-optimal solution visualization, and useful guidance for engineers and practitioners.

### 1.2. Research Objectives

The main aims of the research are as follows:

1. To develop efficient machine learning models that can accurately forecast the compressive and splitting tensile strengths of concrete reinforced with basalt fibers.
2. To optimize model performance through evolutionary hyperparameter optimization with the help of GA, PSO, and DE.
3. To systematically compare a large number of ML-optimization combinations and to select the most successful predictive model.
4. To optimize the composition of BFRC blends to achieve the best performance objectives via a multi-objective optimization model that takes the opposing mechanical performance objectives into account.
5. To interpret model forecasts through explainable ML methods and uncover the strongest parameter mix of BFRC mixes.
6. To convert the proposed framework into a practical engineering tool by developing a graphical user interface.

In this study, compressive strength (CS) and splitting tensile strength (STS) were selected as target properties because they are the most commonly reported mechanical parameters in BFRC studies and provide essential information on load-bearing capacity, matrix strength, and crack resistance. Although shear strength is also important for structural applications, the availability of consistent experimental shear strength data is limited compared with that of CS and STS datasets. Therefore, CS and STS were considered to ensure reliable machine learning model development.

The remainder of this manuscript is organized as follows. Section 2 presents the literature review on machine learning-based concrete strength prediction, evolutionary optimization techniques, multi-objective mix design, and explainable artificial intelligence approaches. Section 3 describes the proposed methodology, including the machine learning models, evolutionary hyperparameter optimization algorithms, NSGA-II-based mix optimization, SHAP analysis, and GUI development. Section 4 presents the experimental setup, including dataset characteristics, model implementation details, and evaluation criteria. Section 5 discusses the results and performance analysis of the developed framework, while Section 6 provides an in-depth discussion of the findings, engineering implications, and limitations of this study. Finally, Section 7 concludes the manuscript by summarizing the major contributions and highlighting future research directions.

## 2. Literature Review

The process of predicting and designing fiber-reinforced concrete, particularly BFRC, has undergone significant change due to the most recent advancements in machine learning and optimization techniques. In recent years, BFRC’s superior tensile strength, durability, and sustainability attributes have drawn increased attention from researchers.

The mechanical performance of concrete, fiber-reinforced concrete (FRC), and BFRC is commonly evaluated using standardized testing procedures. Compressive strength is determined according to ASTM C39/C39M [11], while splitting tensile strength is measured following ASTM C496/C496M [12]. These tests are widely used to assess the influence of fiber characteristics on the mechanical properties of FRC and BFRC, particularly compressive and tensile strength.

### 2.1. Machine Learning-Based Prediction of BFRC Mechanical Properties

Several representative studies have employed machine learning approaches to predict the mechanical properties of BFRC. Besides machine learning studies, recent investigations have shown that microscopic material interactions, including the behavior of the water film and cement matrix, significantly influence the mechanical performance of concrete materials [33]. The following discussion summarizes these studies, highlighting the advantages of ensemble learning methods over conventional regression models for capturing nonlinear material behavior. Hybrid machine learning models have been developed to predict the tensile and compressive strengths of sustainable BFRC [34]. Furthermore, multi-output machine learning models have been proposed to simultaneously predict compressive, splitting tensile, and flexural strengths, demonstrating the benefits of joint learning using correlated mechanical properties [35]. Ensemble and tree-based machine learning models have gained considerable attention because of their robustness and interpretability. Ensemble machine learning methods combined with SHAP analysis have been employed to predict the splitting tensile strength of BFRC, providing valuable insights into feature importance and nonlinear relationships [36]. Hybrid recurrent neural networks have also been applied to improve prediction accuracy by modeling freeze–thaw damage effects in BFRC, highlighting the influence of environmental degradation on mechanical performance [37]. Optimized machine learning methods have further been extended to generalized fiber-reinforced concrete systems. Hyperparameter-optimized machine learning models have demonstrated high effectiveness in predicting the performance of various fiber types, including basalt fibers [38]. The application of explainable machine learning models for predicting the strength properties of BFRC further emphasizes the importance of transparency and interpretability in engineering prediction models [39]. In addition, ensemble learning approaches have demonstrated that advanced hybrid machine learning models can effectively predict the compressive and tensile strengths of BFRC [40]. Machine learning has also been successfully applied to predict the mechanical properties of chopped- and minibar-reinforced high-performance concrete, demonstrating that data-driven models can generalize across different reinforcement configurations and effectively capture complex concrete behavior [41]. Recent studies have also investigated the mechanical behavior and structural performance of advanced concrete systems under different loading conditions. Experimental investigations have demonstrated that reinforcement configuration, composite action, and material characteristics significantly influence the strength and deformation behavior of concrete structures [42,43,44]. In addition, the fatigue behavior and shear performance of fiber-reinforced concrete members have been successfully modelled using analytical and experimental approaches, providing valuable insights into the structural response of fiber-reinforced cementitious composites [45,46].

### 2.2. Metaheuristic Optimization for ML Model Enhancement

Although the use of ML models is associated with high predictive power, the optimal performance is conditional on the selection of hyperparameters. Recent studies have further demonstrated that machine learning models integrated with optimization techniques can substantially improve prediction accuracy and model robustness in complex engineering applications [47]. To solve this problem, scholars have increasingly focused on the use of metaheuristic optimization methods. Metaheuristic-optimized machine learning models have been applied to enhance the prediction reliability of concrete structures, demonstrating better convergence characteristics than conventional grid-based tuning strategies [48]. In addition, high-strength concrete prediction models have been optimized using explainable machine learning and nature-inspired algorithms, resulting in improved model accuracy and transparency [49]. Nevertheless, the available research primarily relies on a single optimization algorithm, which may limit model robustness and adaptability across different datasets. Comparative studies employing multiple evolutionary optimizers, such as Genetic Algorithm (GA), Particle Swarm Optimization (PSO), and Differential Evolution (DE), remain limited, particularly for BFRC-specific applications. Besides prediction accuracy, constitutive modeling and material behavior have also received considerable attention in computational concrete research. Advanced elastoplastic and damage constitutive models have improved the numerical simulation of concrete behavior under complex loading conditions and have provided reliable benchmark data for data-driven prediction methods [50,51]. Furthermore, theoretical prediction models for recycled aggregate concrete have demonstrated the importance of accurately representing material characteristics when developing robust predictive frameworks [52].

### 2.3. Multi-Objective Optimization of Concrete Mix Design

Concrete mix design involves balancing multiple conflicting objectives, including maximizing mechanical strength while minimizing material consumption, cost, and environmental impact. A machine learning-based multi-objective framework for concrete mix design has demonstrated the effectiveness of the NSGA-II algorithm in identifying Pareto-optimal solutions [53]. Similarly, machine learning integrated with NSGA-II has been applied to optimize the performance objectives of recycled aggregate concrete [54]. Furthermore, an explainable machine learning-based multi-objective optimization framework incorporating Pareto front analysis and SHAP-based interpretation has been developed for nanosilica-modified concrete, providing improved optimization transparency and decision support [55]. These studies demonstrate that ML integrated with NSGA-II is effective for multi-objective concrete mix optimization. However, they provide limited emphasis on comparative evolutionary hyperparameter optimization, model interpretability, and practical engineering deployment. The proposed framework addresses these gaps by integrating GA, PSO, and DE for hyperparameter optimization, SHAP-based explainable AI, and GUI-based decision support within a unified BFRC optimization framework.

### 2.4. Explainable AI and Practical Decision Support

Explainable artificial intelligence (XAI) has become an important component for improving the acceptance and trustworthiness of machine learning models in civil engineering applications. SHAP analysis has been demonstrated to be effective in identifying the most influential BFRC mix parameters and capturing nonlinear feature interactions [36,39]. In addition, interpretable and practice-oriented machine learning models have been shown to improve the reliability and applicability of hybrid fiber-reinforced recycled concrete prediction systems [56]. Recent studies have further demonstrated that advanced cementitious composites can simultaneously improve mechanical performance and long-term durability through optimized material design. The incorporation of supplementary cementitious materials and optimized fiber reinforcement has been shown to enhance strength development and structural reliability under different service conditions [57]. These findings further support the development of intelligent prediction frameworks for optimizing the performance of advanced concrete materials. However, existing studies have primarily employed SHAP as a model interpretation technique without integrating it with evolutionary optimization and practical deployment tools. To address this limitation, the proposed framework incorporates SHAP-based explainable AI within an integrated workflow that combines evolutionary hyperparameter optimization and GUI-based decision support, thereby improving the transparency and practical usability of BFRC mix design.

### 2.5. Research Gaps

The following research gaps were identified through a critical analysis of the literature:

1. Inadequate comparative analysis of ML models: the majority of studies use one machine learning model that does not involve systematic comparisons of RF, GBR, and XGBoost to predict the strength of BFRC.
2. Limited evolutionary hyperparameter optimization: existing studies typically focus on a single optimizer and rarely compare GA, PSO, and DE in terms of robustness and convergence speed.
3. Separation of prediction and mix design optimization: the traditional approach taken in the literature focuses on either strength prediction or mix optimization, but few studies attempt to combine such work into a single workflow.
4. Lack of multi-objective optimization of BFRC: only a few studies apply NSGA-II to BFRC to simultaneously optimize compressive and splitting tensile strengths.
5. Absence of practical deployment tools: the majority of the models are just theoretical, and little emphasis is placed on explainability-based decision-support systems or GUI-based implementation to be used in real-life engineering applications.

Building upon previous studies that have successfully combined machine learning with NSGA-II for concrete optimization, the present study extends the existing body of knowledge by integrating comparative evolutionary hyperparameter optimization, explainable artificial intelligence, and GUI-based engineering deployment into a unified decision-support framework specifically developed for BFRC. Specifically, GA, PSO, and DE are systematically compared for hyperparameter optimization to improve prediction performance and reduce the bias associated with manual tuning. The optimized models are subsequently incorporated into an NSGA-II-based multi-objective optimization framework to generate Pareto-optimal BFRC mix designs. Furthermore, SHAP-based explainable AI enhances model transparency, while a GUI enables practical real-time engineering decision support.

## 3. Methodology

**Figure 1:**
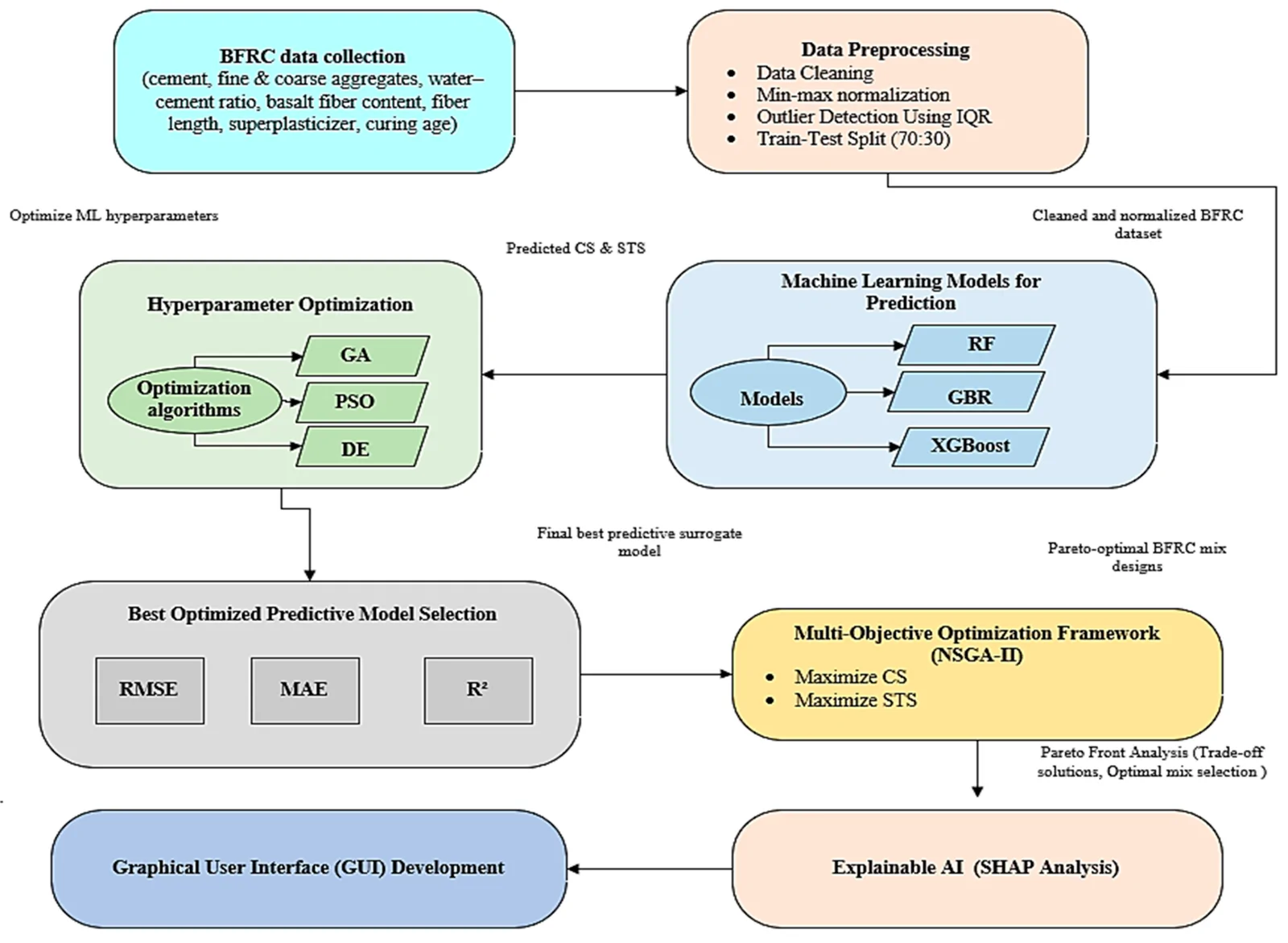
Overall workflow chart for the proposed evolutionary ML-based multi-objective optimization framework for BFRC.

Figure 1 illustrates the workflow of the proposed multi-objective optimization framework based on evolutionary ML. The experimental data on the BFRC mix compositions and the relevant mechanical properties were gathered from published and authenticated academic sources. To enhance model learning efficacy and reliability, the collected data were pre-processed systematically through data cleaning, normalization, and train-test partitioning. Thereafter, several machine learning-based regression models were formed to forecast CS and STS. Evolutionary hyperparameter optimization methods, such as GA, PSO, and DE, were used to improve their predictive performance and provide better convergence and generalization. This step was followed by the application of the NSGA-II multi-objective optimization model to produce Pareto-optimal BFRC mix designs, enabling the simultaneous optimization of CS and STS with performance trade-offs. XAI analysis using SHAP was performed in order to measure the effects of the individual mix parameters on the strength predictions to ensure transparency and engineering interpretability. Finally, the developed optimized predictive and optimization models were integrated into a GUI to provide engineers with real-time strength prediction and practical decision support.

### 3.1. Random Forest Regressor (RF)

In this study, the RF uses bagging to train multiple decision trees on random data subsets while selecting random features at each split, reducing variance and overfitting, while improving model diversity. The input layer receives the BFRC mix design and fiber-related parameters, including cement content, fine and coarse aggregates, water–cement ratio, basalt fiber dosage, fiber length, superplasticizer content, and curing age. Based on the original training set (X,Y), several bootstrap samples are created by random sampling (with replacement). The bootstrap sets have the same size as the original training set but contain duplicated observations. Each bootstrap sample is trained using an independent decision tree. A random subsample of input features is tested at each internal node of a tree on whether to split or not, making the trees less similar and less correlated. All prediction trees are trained and the results are averaged arithmetically. The final results obtained are the forecasted CS and STS of BFRC.

### 3.2. Gradient Boosting Regressor (GBR)

The GBR used in the research is based on a layered learning architecture that aims to successively increase the accuracy of the prediction by progressive error correction. First, the input layer is fed with normalized BFRC mix design and fiber-related parameters, including cement content, proportions of aggregates, water–cement ratio, basalt fiber volume fraction, fiber length, superplasticizer dosage, and curing age. An initial prediction layer generates a baseline estimate, typically the mean of the target outputs. Residuals are then computed as the difference between experimental values and predictions, and sequential decision trees are trained to learn these errors. A learning rate (shrinkage) controls each tree’s contribution, and all learners are aggregated to produce the final predictive model. Lastly, the estimated CS and STS of basalt fiber-reinforced concrete are generated in the output layer.

GBR effectively handles the bias–variance trade-off through sequential error correction, making it suitable for modeling the nonlinear and heterogeneous behavior of BFRC. It captures complex interactions among mix constituents and delivers high predictive accuracy, particularly for small- to medium-sized datasets. The model also supports flexible optimization across various loss functions, enhancing its adaptability. Overall, GBR is a powerful and efficient tool for predicting the compressive and splitting tensile strengths of basalt fiber-reinforced concrete.

### 3.3. Extreme Gradient Boosting (XGBoost)

The XGBoost regression model developed in this research represents a layered structure that effectively projects the nonlinear correlation between the variables of the BFRC mix design and mechanical properties. Normalized inputs, including cement and water content, aggregate proportions, superplasticizer dosage, and basalt fiber characteristics, are first processed to generate an initial baseline prediction. A prediction layer is built to produce an initial forecast that mainly reflects the mean of the required mechanical properties. The regularized tree construction layer constructs regression trees sequentially through minimization of a regularized objective function that penalizes tree complexity and excessively high leaf weights, thereby improving generalization. The additive ensemble layer then incorporates each additional trained tree to the current ensemble with a learning rate to regulate model updates and avoid overfitting. The last step is the output layer, which generates the optimized forecasts of CS and STS of basalt fiber-reinforced concrete.

XGBoost has built-in regularization processes that effectively regulate the complexity of the models, thereby minimizing the risk of overfitting to learn on heterogeneous experimental data. In addition, XGBoost can work with sparse and non-uniform data distributions (typically encountered in experimentally obtained BFRC datasets). Due to its capacity to precisely model highly nonlinear relations among the mix composition, the properties of fibers, and the resulting mechanical performance, XGBoost has the potential to attain high predictive accuracy for compressive strength and splitting tensile strength. Such properties render XGBoost a powerful and dependable predictor of the BFRC strength evaluation and optimization.

**Model Output Representation:** For all developed ML models, namely RF, GBR, and XGBoost, the predicted output is represented in vector form as:

(1) $$Yˆ=\left[CSˆ\;STSˆ\right]$$

where CSˆ denotes the predicted compressive strength (MPa), and STSˆ represents the predicted splitting tensile strength (MPa) of BFRC. Each machine learning model learns a nonlinear mapping between the normalized input feature vector $X\in R^{10}$ and the output strength properties, expressed as:

(2) $$Yˆ=f_{ML}(X)$$

where $f_{ML}(\cdot )$ corresponds to the trained RF, GBR, or XGBoost model.

While individual ML models provide accurate predictions for compressive and splitting tensile strengths (MPa) independently, practical BFRC mix design requires simultaneous enhancement of multiple mechanical properties. Therefore, the predicted outputs CSˆ and STSˆ are jointly considered as objective functions within an evolutionary optimization framework.

The formulation of the multi-objective optimization problem is as follows:

(3) Maximize {CSˆ(X) STSˆ(X)

where $X_{min}$ and $X_{max}$ represent the lower and upper bounds of the input variables, including material contents ($kg/m^{3}$), fiber length (mm), fiber diameter (mm), fiber volume fraction (%), and curing age (days).

The predicted outputs generated by the machine learning models serve as fitness evaluation functions within the evolutionary optimization process. Three population-based evolutionary algorithms are employed, GA, PSO, DE. Each evolutionary algorithm iteratively updates candidate solutions (BFRC mix designs) by optimizing the predicted output vector Yˆ.

The fitness function for optimization is defined as:

(4) F(X)=[CSˆ(X),STˆS(X)]

where $\hat{CS}(X)$ and $\hat{STS}(X)$ are the predicted compressive strength and splitting tensile strength, respectively, expressed in MPa.

Selection, crossover, mutation (GA), velocity and position updates (PSO), and vector mutation and recombination (DE) are some of the evolutionary operators that are used to search the space and find the best mix configurations of BFRC. As CS and STS might be characterized by trade-off behavior, the set of solutions to the optimization problem forms a Pareto-optimal set, with neither objective able to be optimized without worsening the other. These Pareto-front solutions are ideal BFRC mix designs in terms of balancing various mechanical performance criteria. The optimized output vectors from the evolutionary algorithms are integrated into a GUI that enables users to visualize predicted CS and STS, compare Pareto-optimal BFRC mix designs, and select the most suitable solutions based on engineering requirements.

### 3.4. Hyperparameter Optimization

Given the strong influence of hyperparameters on ensemble ML performance and the possibility of high computational cost and local optima in complex, nonlinear, high-dimensional problems such as those encountered in BFRC, this study employs evolutionary optimization for automated global tuning. Specifically, population-based algorithms (GA, PSO, and DE) are used to optimize the hyperparameters of RF, GBR, and XGBoost models.

#### 3.4.1. Hyperparameter Optimization Problem Formulation

Let the hyperparameter vector of a machine learning model be defined as:

(5) $$\theta =\left[\theta_{1},\theta_{2},\ldots ,\theta_{k}\right]$$

where $\theta_{k}$ denotes the $k^{th}$ tunable hyperparameter (dimensionless). The goal of optimization is to determine the ideal set of hyperparameters $\theta^{*}$ that minimizes prediction error while maximizing model generalization. The optimization problem is formulated as:

(6) $$\theta^{*}=arg\;\underset{\theta }{min}F(\theta )$$

K-fold cross-validation is used for evaluation in order to ensure strong generalization and avoid overfitting.

#### 3.4.2. Genetic Algorithm (GA)

In hyperparameter optimization, GA searches the search space by mutating a population of candidate solutions, each of which represents a potential hyperparameter setting of the ML model. It constantly employs selection, crossover, and mutation operators to avoid premature convergence and balances exploration and exploitation to identify near-optimal solutions.

#### 3.4.3. Particle Swarm Optimization (PSO)

PSO is computationally efficient and ideally applicable to continuous hyperparameter tuning problems, unlike evolutionary algorithms that are based on the use of genetic operators. PSO-based optimization allows particles to navigate the search space by modifying their location and velocity according to both individual experience and global knowledge. PSO offers fast convergence, low computational cost, and strong global search capability, making it well suited for optimizing the hyperparameters of ensemble models such as RF, GBR, and XGBoost, particularly for nonlinear and noisy BFRC data.

#### 3.4.4. Differential Evolution (DE)

DE is an effective population-based evolutionary algorithm that is particularly aimed at continuous and real-valued optimization. Due to its straightforward architecture, excellent global search performance, and rapid convergence behavior, DE is especially useful for optimizing ML hyperparameters in nonlinear, high-dimensional search problems encountered in BFRC strength prediction models. In DE, all members of the population represent candidate hyperparameter vectors of the ML model. The model is presented as optimally represented.

#### 3.4.5. Optimized Model Representation

The ML models are retrained using the ideal hyperparameter configuration derived using GA, PSO, or DE, following the evolutionary hyperparameter optimization procedure. The optimized models exhibit enhanced learning capability, reduced generalization error, and improved robustness against experimental noise compared to their non-optimized counterparts. Evolutionary hyperparameter tuning offers clear advantages over traditional methods, including global search capability, reduced risk of local optima, adaptive tuning, robustness to noisy data, and scalability to complex ensemble models. These strengths make it highly effective for modeling nonlinear relationships in BFRC, while the optimized models provide a strong basis for multi-objective optimization of CS and STS under practical constraints.

### 3.5. Multi-Objective Optimization Framework (NSGA-II with Pareto Front Analysis)

The current study employs a multi-objective optimization framework using NSGA-II with Pareto front analysis to simultaneously optimize the CS and STS of BFRC under realistic design constraints. NSGA-II is employed in this study due to its ability to efficiently handle multi-objective optimization problems with conflicting objectives. Its key features, including elitism, fast non-dominated sorting, and crowding-distance-based diversity preservation, make it particularly suitable for optimizing BFRC mix designs. NSGA-II organizes candidate solutions into successive Pareto fronts ($F_{1},F_{2},\ldots$), where the first front ($F_{1}$) consists of the non-dominated solutions that represent the best trade-offs between objectives. To maintain solution diversity along the Pareto front and prevent clustering, a crowding distance metric is calculated for each solution. In addition, with elitist selection, both parent and offspring populations are taken into account in the evolutionary process, and the solutions that can perform the best are retained across generations, and a vigorous search of the design space is possible. The Pareto front is the set of solutions that are not dominated by any other solution and that represent an optimal trade-off between compressive and tensile strengths. The proposed framework incorporates the optimized machine learning models (RF, GBR, and XGBoost) as surrogate fitness evaluators to effectively forecast the mechanical properties of BFRC for each candidate mix design.

### 3.6. Explainable Artificial Intelligence (SHAP Analysis)

Although the optimized machine learning models demonstrate high predictive accuracy for estimating the mechanical properties of BFRC, their ensemble and boosting-based structures inherently behave as black-box models. XAI approaches using SHAP were employed to improve engineering trustworthiness, interpretability, and transparency. SHAP is a game-theoretic method that equitably assigns each input feature’s contribution to the final model output to explain individual predictions. A higher expected strength is shown by a positive SHAP score, whilst a lower influence is indicated by a negative number. 

The best-performing optimized ML model was further analyzed using SHAP to determine the effects of the BFRC mix design variables, including cement content, water–cement ratio, basalt fiber volume fraction, fiber length, aggregate proportions, superplasticizer dosage, and curing age. The features of input data were ranked by their overall contribution to CS and STS using SHAP summary plots. Nonlinear relationships between individual mix parameters and the predicted outputs were analyzed using dependence plots to evaluate the effects of the interaction between the two. Additionally, impact plots were used to visualize the direction of feature impact and the magnitude of feature impact across the dataset was employed. The explainability framework allows SHAP to provide meaningful engineering information on the complicated nonlinear response of BFRC, discover superior parameters that control the mechanical performance, and confirm the physical consistency of the optimized ML predictions. Such interpretability improves confidence in the proposed AI-based modeling framework and contributes to its implementation in real-life civil engineering solutions.

### 3.7. Graphical User Interface (GUI) Development

The optimized predictive and multi-objective optimization framework was embedded in a standalone GUI to enable its practical implementation and accessibility to users. The main goal of the GUI is to transform the suggested artificial intelligence (AI)-based modeling and optimization strategy into an easy-to-use decision-support system that would be accessible to engineers, researchers, and practitioners without the need to be knowledgeable in ML or coding. The GUI supports a logical process that involves receiving user input, preprocessing data, optimizing machine learning prediction, and visualizing the results. First, the BFRC mix design parameters are entered through intuitive input fields by the user. These inputs are automatically scaled and pre-treated using the same procedures applied during model training. The data is then processed and input into the optimized ML model to provide real-time predictions of CS and STS. Besides prediction, the GUI also includes the multi-objective optimization results that were obtained with the NSGA-II framework. Pareto-optimal BFRC mix designs are offered that users can interactively explore as representing trade-offs between competing mechanical goals. This facilitates informed decision-making in order to have different mix composition choices, which would then be compared, and the best solutions are then selected depending on the performance requirements and practical limitations. The resultant GUI effectively bridges the gap between current techniques of advanced AI artificial intelligence and practical civil engineering applications, providing a transparent, efficient, and interactive platform to test and optimize BFRC mix design. The given integration increases the practical value of the proposed framework and makes it applicable for use in research and industrial environments.

## 4. Experimental Setup

In this section, the BFRC dataset, preprocessing, computing environment, hyperparameter optimization policy, and the evaluation metrics are described for the design, training, and testing of the proposed evolutionary machine learning framework and for strength prediction.

### 4.1. Dataset Overview

The data used in this study were compiled through a comprehensive review of previously published journal articles and doctoral dissertations related to BFRC. To ensure consistency and comparability, only experimental results from specimens tested at a curing age of 28 days were considered, as 28-day compressive strength is widely recognized as a standard indicator of structural concrete performance. The dataset was obtained from an openly available benchmark source on Mendeley Data [58], which has been adopted in previous BFRC machine learning studies [34,35,36,37,38,39,40], supporting the reliability and reproducibility of the analysis. The final dataset consisted of 270 samples for CS prediction and 267 samples for STS prediction. Although the dataset size is moderate, it is comparable to those used in previous BFRC machine learning studies and is sufficient for developing and validating reliable machine learning models for BFRC strength prediction. Each data sample included 10 input variables representing mix composition and fiber characteristics, while the output variables represented the corresponding mechanical properties of BFRC. The broad range of the material proportions and fiber configurations indicates the different experimental conditions, allowing for robust and general machine learning development.

The collected BFRC dataset was compiled from multiple experimental studies reported in the literature, incorporating diverse concrete mix designs and testing conditions. The dataset includes variations in aggregate types, cement composition, supplementary additives, water–cement ratios, and basalt fiber characteristics to represent the practical diversity of BFRC mixtures. The basalt fibers considered in the dataset exhibited different geometric properties, with fiber lengths ranging from 6–30 mm, diameters ranging from 0.013–0.020 mm, and varying fiber volume fractions depending on the mixture design adopted in each experimental study. Although several studies reported important fiber-related parameters, uniform mechanical properties of basalt fibers, such as tensile strength and elastic modulus, were not consistently available across all sources; therefore, these parameters were not included as input variables in the present modeling framework.

Reliable model development was supported by considering only datasets with standardized mechanical testing conditions, particularly 28-day curing strength measurements, which represent the most commonly adopted evaluation period for concrete performance assessment. Despite variations in mixture proportions and material constituents among different studies, all samples were treated as comparable based on their consistent testing methodology and reported mechanical responses. The final dataset covered a broad range of mechanical performance, enabling the proposed machine learning models to learn complex nonlinear relationships between input mixture parameters and BFRC strength characteristics. Specifically, the compressive strength values ranged from 22.8 to 89.6 MPa, with an average value of 55.84 MPa, while the splitting tensile strength values varied from 2.12 to 8.94 MPa, with a mean value of 5.36 MPa. This wide variation in strength levels enhances the diversity of the dataset and supports the development of robust predictive models applicable to different BFRC mix design scenarios.

Table 1 summarizes the variables used for developing the BFRC prediction models. The dataset consists of 10 input variables representing the concrete mix composition and fiber characteristics, along with 3 output parameters describing the mechanical properties. These variables constitute the input and target features used for machine learning model development and evaluation.

#### Input and Output Variables

This dataset consists of 13 parameters, of which 10 are input variables (features), which include the mix composition and fiber properties, and three output variables (targets), which are the mechanical properties of BFRC. The input parameters make both the traditional concrete constituents and the basalt fiber properties. The mathematical representation input variable vector

(7) $$X=\left[C,SF,FA,CA,FA_{g},W,SP,FD,FL,FF\right]$$

where SF represents the silica fume content, C represents the cement content, FA denotes the fly ash content, $FA_{g}$ represents the fine aggregate content, CA represents the coarse aggregate content, W represents the water content, SP represents the water-reducing agent (kg/m^3^), FD represents the basalt fiber diameter (mm), FL represents the basalt fiber length (mm), FF represents the basalt fiber volume fraction (%). The target variables used to measure the mechanical performance of BFRC were the following:

(8) Y=[CS,STS]

where CS refers to compressive strength, and STS refers to splitting tensile strength (STS, MPa).

The dataset was compiled from peer-reviewed studies and graduate research conducted across multiple universities, where experiments were performed under expert supervision using standard materials and established laboratory procedures. This ensured the reliability and scientific validity of the data. A key strength of the dataset is its diversity, as it includes results from different regions, materials, and experimental conditions, enhancing its robustness and generalizability. To analyze the data, this study employed ML models with ten input variables describing mix composition. Unlike traditional regression methods, which may face issues like multicollinearity and reduced reliability, ML algorithms effectively capture complex nonlinear relationships, resulting in improved predictive accuracy and robustness for modeling concrete mechanical properties.

### 4.2. Data Preparation

To investigate the distribution, variability, and reliability of the collected data, a comprehensive statistical analysis was conducted. The dataset exhibited substantial diversity across all input parameters, reflecting the integration of BFRC mix designs obtained under different experimental conditions. The diversity of the dataset is also reflected in the output variables. The compressive strength values ranged from 22.8 MPa to 89.6 MPa, with an average value of 55.84 MPa, while the splitting tensile strength values ranged from 2.12 MPa to 8.94 MPa, with an average value of 5.36 MPa. These wide ranges and average values indicate that the dataset includes BFRC mixtures with diverse mechanical performance characteristics, enhancing the robustness, applicability, and generalization capability of the developed machine learning models across a broad spectrum of mixture compositions and performance conditions. Considering the nonhomogeneous nature of the data compiled from multiple experimental studies, a systematic preprocessing pipeline was implemented to improve data quality and learning efficiency. The preprocessing procedure included data cleaning, normalization, outlier detection, and dataset partitioning. Initially, the dataset was examined for missing values, duplicated records, and inconsistencies in measurement units. Samples containing missing output values were removed to avoid bias in model training and evaluation. To ensure consistency, all material quantities were standardized to kg/m^3^, fiber diameters were converted to millimeters (mm), and mechanical strength properties were expressed in MPa. As the numerical ranges of the input variables varied considerably (e.g., cement content, water content, and fiber dimensions), feature scaling was required to prevent variables with larger magnitudes from dominating the learning process. Therefore, all input features were normalized using min–max scaling.

Following normalization, outlier detection was performed to identify and remove extreme observations that could arise from experimental variability or inconsistencies in the reported literature data. The interquartile range (IQR) method was adopted because of its robustness and nonparametric nature. Outliers were carefully examined and removed only when they were determined to be physically unrealistic or statistically inconsistent.

To ensure an unbiased assessment of model performance, the cleaned dataset was randomly divided into training and testing subsets. The training dataset was used for model development and hyperparameter optimization, whereas the testing dataset was reserved for independent model evaluation. In addition, tenfold cross-validation was employed during training to improve model generalization and reduce the risk of overfitting. This approach ensured that each data sample contributed to both training and validation, thereby providing a more reliable estimation of model performance.

All experiments were conducted in Python 3.x using Scikit-learn and XGBoost for machine learning model development. Evolutionary optimization algorithms were implemented through Differential Evolution with Adaptive Parameters (DEAPs), PySwarm, and PyGAD libraries. Data preprocessing, analysis, and visualization were performed using NumPy, Pandas, and Matplotlib, while SHAP was utilized to improve model interpretability and feature contribution analysis. The simulations were executed on a Linux- or Windows-based system equipped with an Intel i7 (or equivalent) processor and 16 GB of RAM. Owing to the moderate size of the dataset, GPU acceleration was not required.

### 4.3. Hyperparameter Configuration

The initial default hyperparameter settings were used to train baseline RF, GBR, and XGBoost models to obtain baseline performance. The optimal set of hyperparameters to employ in CS and STS prediction was then ascertained using the evolutionary optimization technique, which comprised GA, PSO, and DE. The number of estimators, the tree’s depth, the learning rate, the subsampling ratio, and the feature selection approach were among the model-critical parameters optimized. All the evolutionary algorithms were repeated by looking at the specified search space and optimizing a fitness function that seeks to minimize the validation error (RMSE) on tenfold cross-validation. This method enabled strong convergence without overfitting and early convergence. Dissimilar optimal hyperparameter values were obtained in CS and STS prediction, representing disparate learning complexities associated with the compressive and tensile behavior of BFRC. The optimal hyperparameter configurations for CS and STS prediction were determined through the evolutionary optimization process using GA, PSO, and DE algorithms. The selection of hyperparameter values was based on minimizing the validation RMSE obtained from tenfold cross-validation while ensuring model robustness and generalization capability. Separate optimization procedures were conducted for CS and STS prediction due to the distinct nonlinear relationships between the BFRC mix parameters and their corresponding mechanical properties. All further performance evaluation, explainability analysis and multi-objective optimization were performed on the final optimized configurations. Table 2 shows the hyperparameter configuration and optimal values for evolutionary-optimized ML models.

### 4.4. Performance Metrics

Model performance was evaluated using three widely accepted regression metrics: $R^{2}$, RMSE, and MAE. In terms of error size, variance allocation, and prediction accuracy, these metrics are complementary to one another. The percentage of the observed data that the model can account for was calculated using $R^{2}$. Its values ranged from 0 to 1, where values closer to 1 denote better prediction performance. RMSE is a measure of the standard deviation of the prediction error and indicates the concentration of the residues around zero. It is also sensitive to outliers because the squared term imposes a greater penalty on larger errors. Small RMSE values represent a good model.

MAE is the mean of absolute deviations between actual and predicted values. In contrast to RMSE, it does not square the error term and thus is less sensitive to large deviations. Lower MAE values indicate better predictive performance.

These measures collectively evaluate the level of prediction, the magnitude of error, and the strength of a model, which give an in-depth assessment of the performance of the model.

## 5. Results and Discussion

The findings are addressed through the context of the data characteristics, performance in prediction, optimization, explainability, and implementation in practice.

### 5.1. Predictive Performance of Machine Learning Models

#### 5.1.1. Compressive Strength Prediction

The RF, GBR, and XGBoost models are compared in terms of their predictive performance of CS on various statistical error measures. These outcomes compare the initial versions of the models with their optimized counterparts to evaluate the efficiency of hyperparameter optimization with GA, PSO, and DE. Table 3 quantitatively summarizes the performance of all baseline and optimized models. This implies that evolutionary hyperparameter optimization leads to significant improvements in model accuracy, especially when it comes to boosting-based models. GBR–GA and GBR-DE become very dependable predictors, whereas RF-based models are not very sensitive to optimization. Overall, the results indicate that the use of evolutionary algorithms alongside gradient-boosting models considerably improves CS prediction of BFRC.

**Figure 2:**
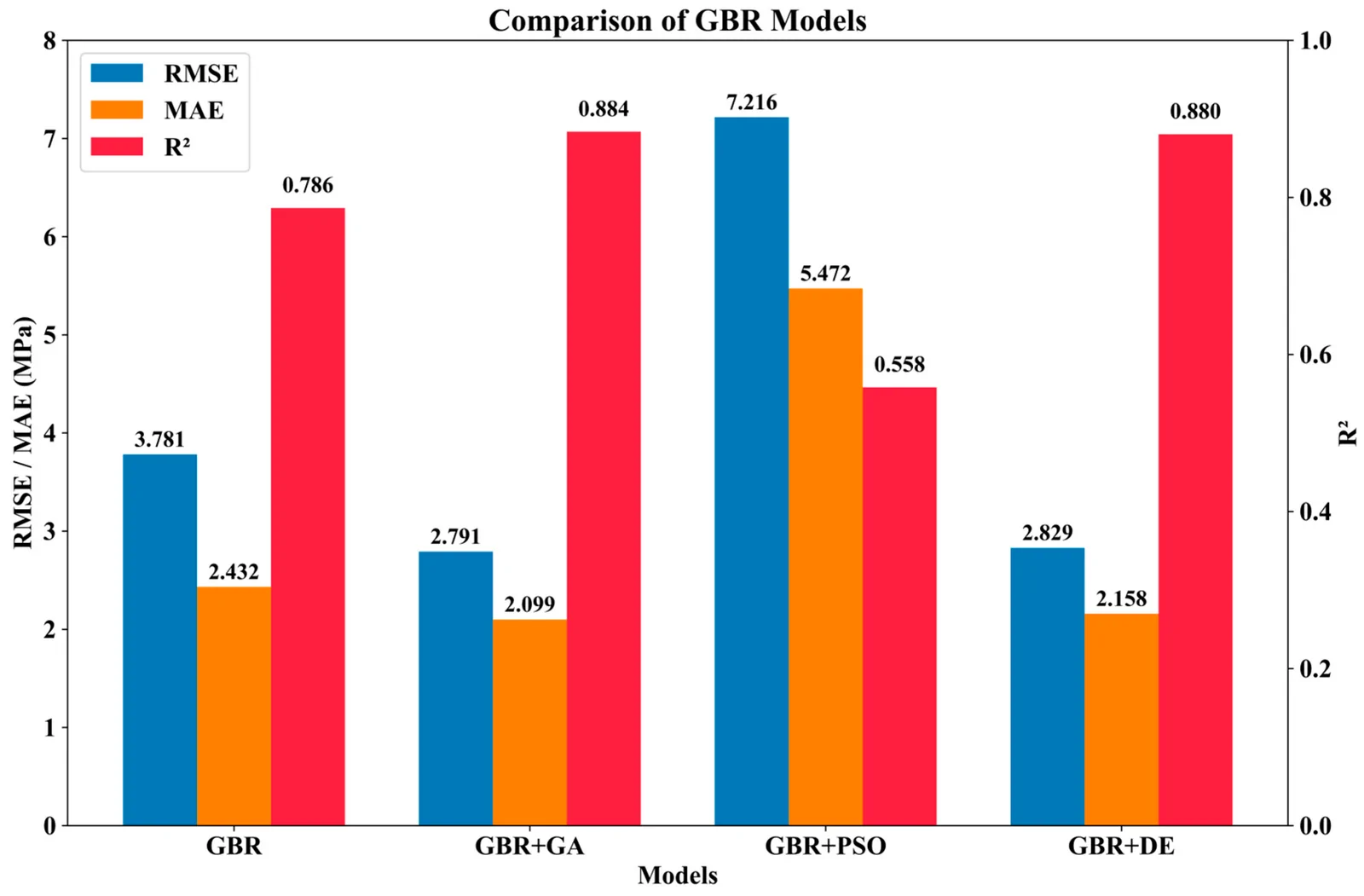
Comparison of error metrics of baseline and evolutionary-optimized GBR models in CS prediction.

**Figure 3:**
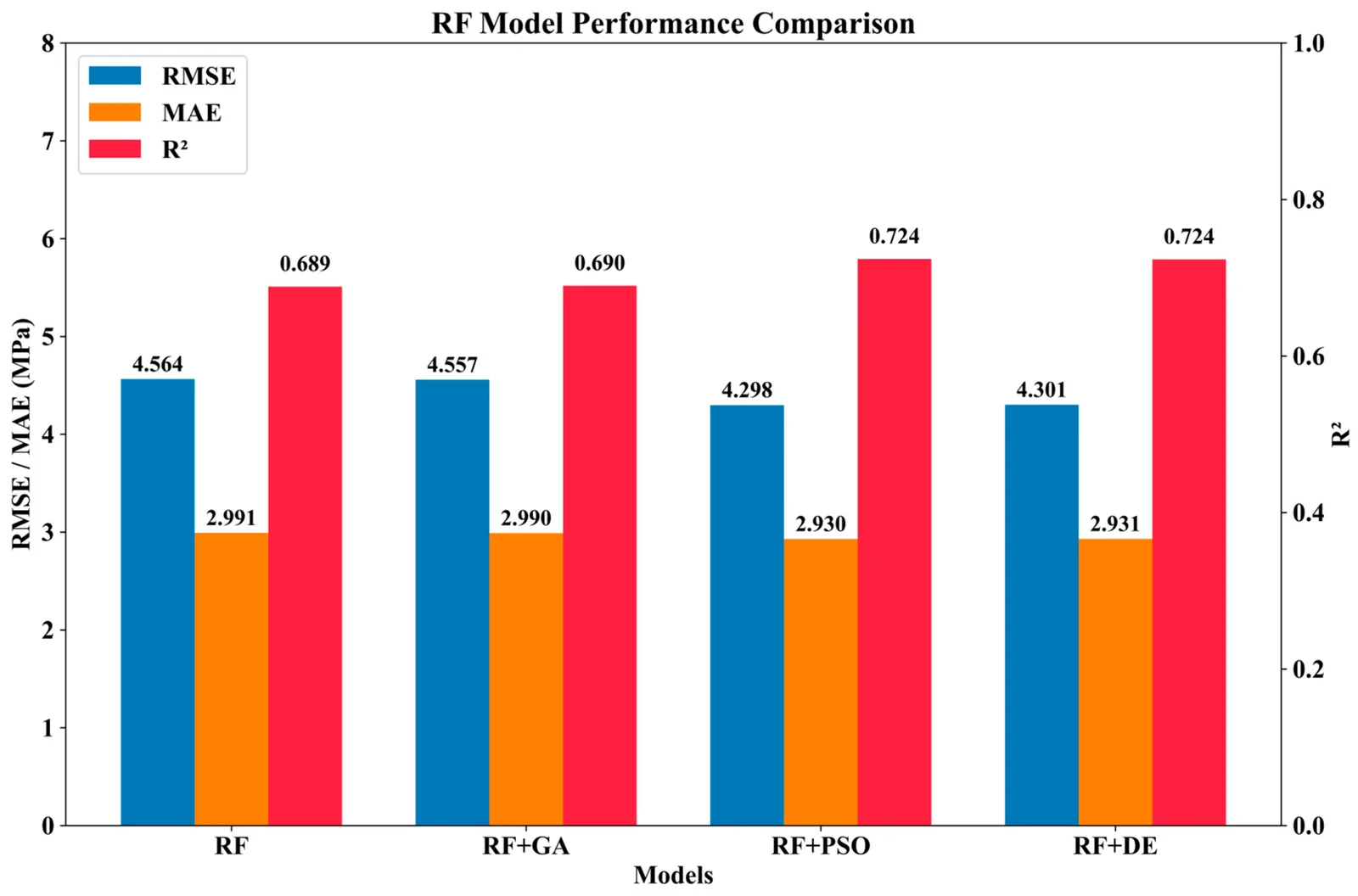
Comparison of baseline and evolutionary-optimized RF models error metrics in CS prediction.

**Figure 4:**
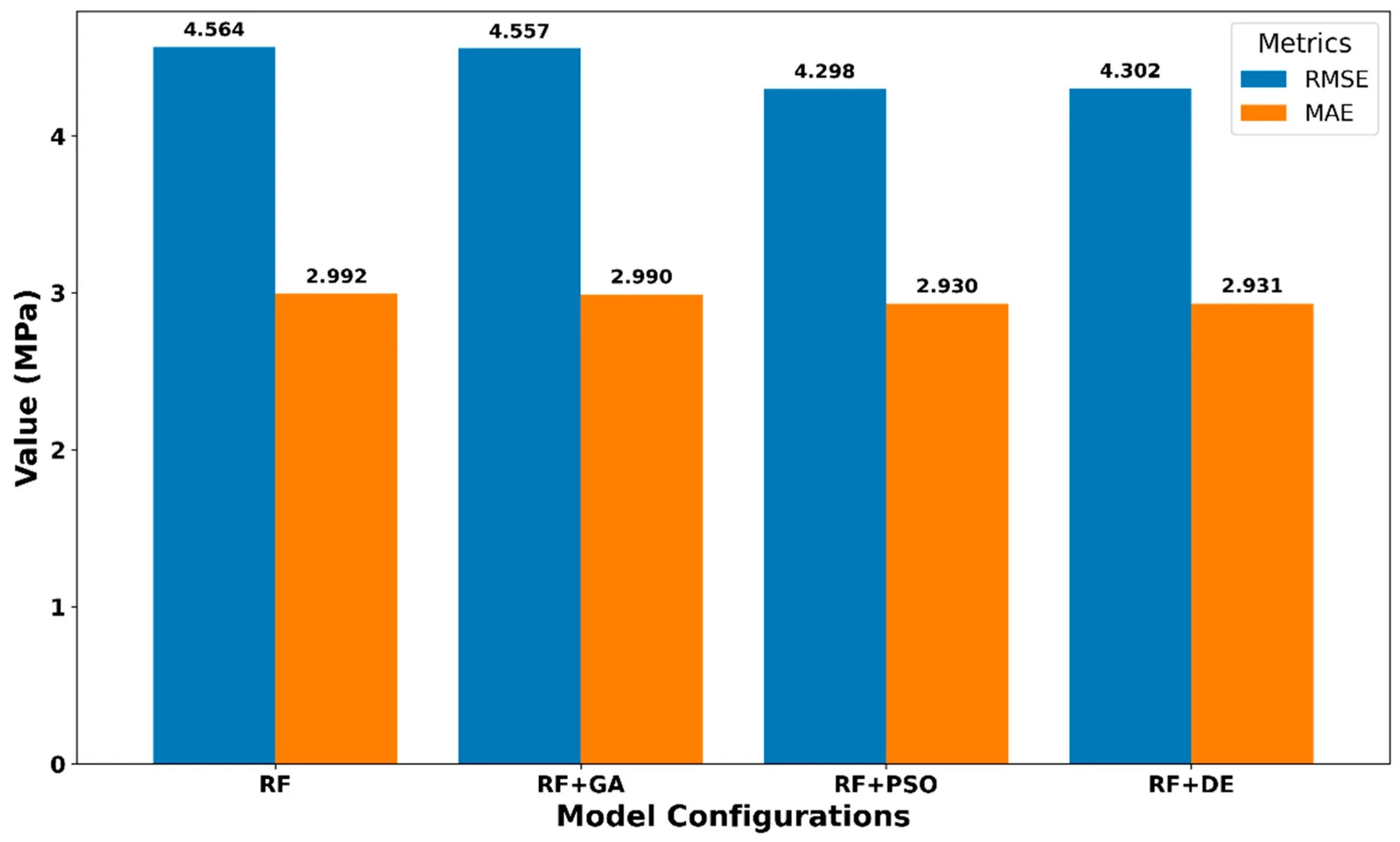
Comparison of the error model for the baseline and evolutionary-optimized XGBoost model in CS prediction.

Figure 2 shows that the baseline GBR achieves moderate accuracy (R^2^ = 0.786, RMSE = 3.78 MPa), while evolutionary optimization significantly improves performance. GBR–GA and GBR-DE achieve higher accuracy (R^2^ ≈ 0.88) with reduced RMSE (~2.8 MPa), whereas GBR-PSO exhibits lower performance (R^2^ = 0.5581, RMSE = 7.216 MPa), which may be attributed to the inability of PSO to identify an optimal hyperparameter configuration for the GBR model under the given search space. The convergence behavior of PSO and the complex nonlinear relationship between BFRC input parameters and compressive strength may have resulted in a suboptimal solution. These findings highlight that the effectiveness of evolutionary optimization depends on the compatibility between the optimizer characteristics and the underlying ML model. Figure 3 indicates that the Random Forest model provides stable but comparatively lower predictive accuracy, with only marginal improvements after evolutionary optimization (R^2^ ≈ 0.72, RMSE ≈ 4.30 MPa, and MAE ≈ 2.93 MPa), reflecting its limited ability to capture the complex nonlinear relationships in BFRC. Figure 4 shows that the evolutionary-optimized XGBoost models achieve the best overall predictive performance, exhibiting higher R^2^ values together with lower RMSE and MAE, thereby demonstrating superior consistency and effectiveness in modeling the highly nonlinear compressive behavior of BFRC.

**Figure 5:**
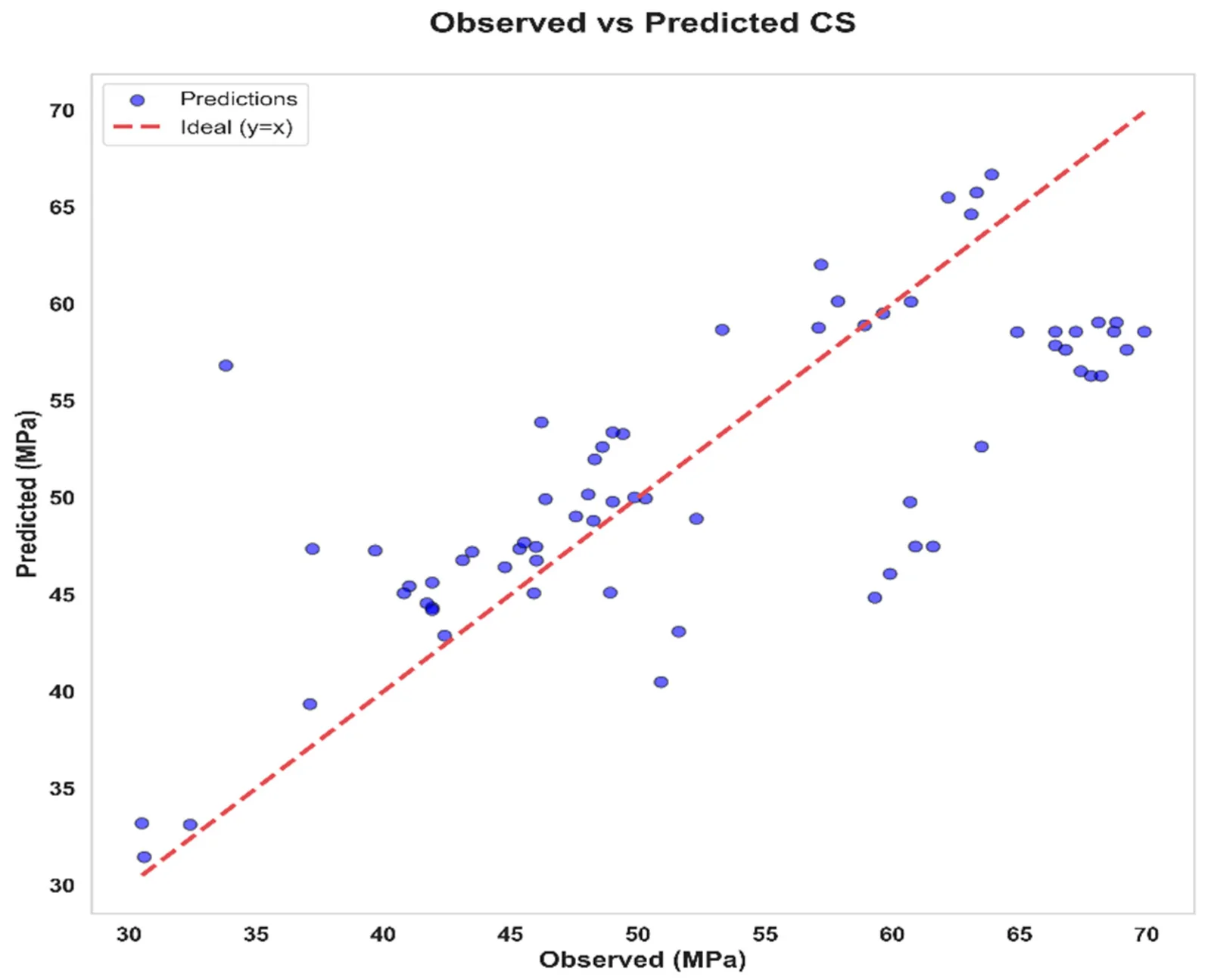
Correlation between observed and predicted CS of BFRC using optimized machine learning models.

Figure 5 illustrates the relationship between the experimentally observed and predicted compressive strength values obtained from the optimized machine learning models. Each data point represents an individual BFRC mix design from the independent test dataset, where the observed compressive strength is plotted against the corresponding predicted value. The data points are closely clustered around the 45° identity line, indicating a high level of agreement between the experimental and predicted values. The regression slope is close to unity with a near-zero intercept, demonstrating minimal systematic bias. Furthermore, the high R^2^ values (>0.90) indicate that more than 90% of the variation in compressive strength is accurately captured using the input variables, including cement content, water content, fiber dosage, and curing age.

Table 4 presents three representative BFRC mix designs covering low, medium, and high compressive strength levels from the test dataset. For each case, the predicted compressive strength closely matches the corresponding experimental value, with absolute errors below 0.70 MPa and relative errors below 2%. These representative examples demonstrate that the optimized machine learning model maintains consistent prediction accuracy across different strength ranges. The results further confirm the reliability and generalization capability of the proposed model for BFRC compressive strength prediction.

#### 5.1.2. Splitting Tensile Strength Prediction

The RF, GBR, and XGBoost models that are used to predict STS are compared using different statistical measures. As compared to compressive strength, STS prediction has a somewhat lower degree of accuracy since tensile testing is more erratic during experimentation. Nevertheless, the evolutionary hyperparameter optimization idea has contributed immensely to the power and precision of any model.

All quantitative comparisons of all the optimized and base models are presented in Table 5. The findings indicate that evolutionary optimization substantially improves model performance, particularly the boosting-based algorithms. The best predictors are GBR-PSO and XGB-PSO, with RMSE values below 0.30 MPa and MAE below 0.20 MPa. Overall, the findings demonstrate that with the integration of evolutionary optimization and advanced ensemble learning techniques, accurate and robust predictions of splitting tensile strength in basalt fiber concrete can be made.

**Figure 6:**
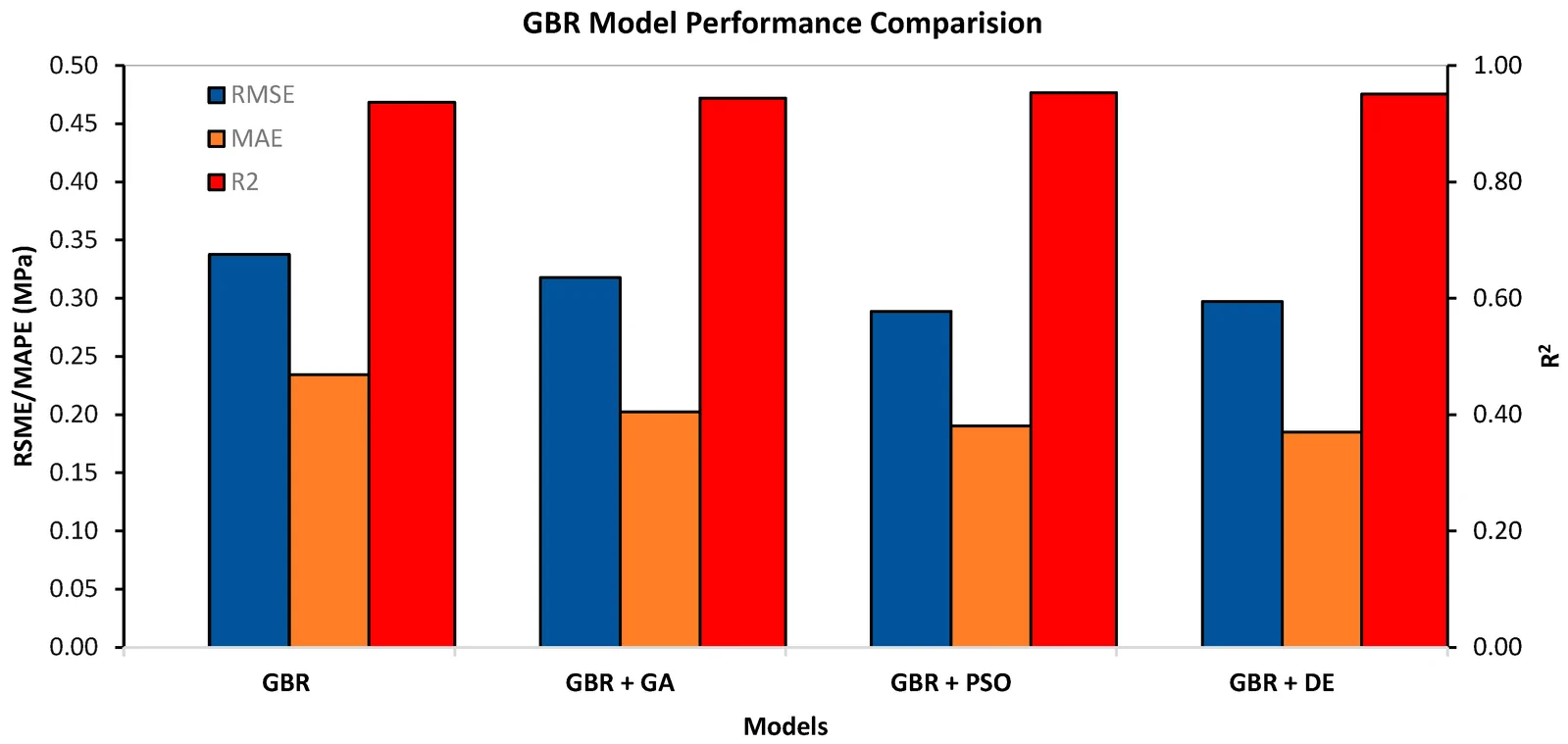
Comparison of error metrics for GBR models in STS prediction.

**Figure 7:**
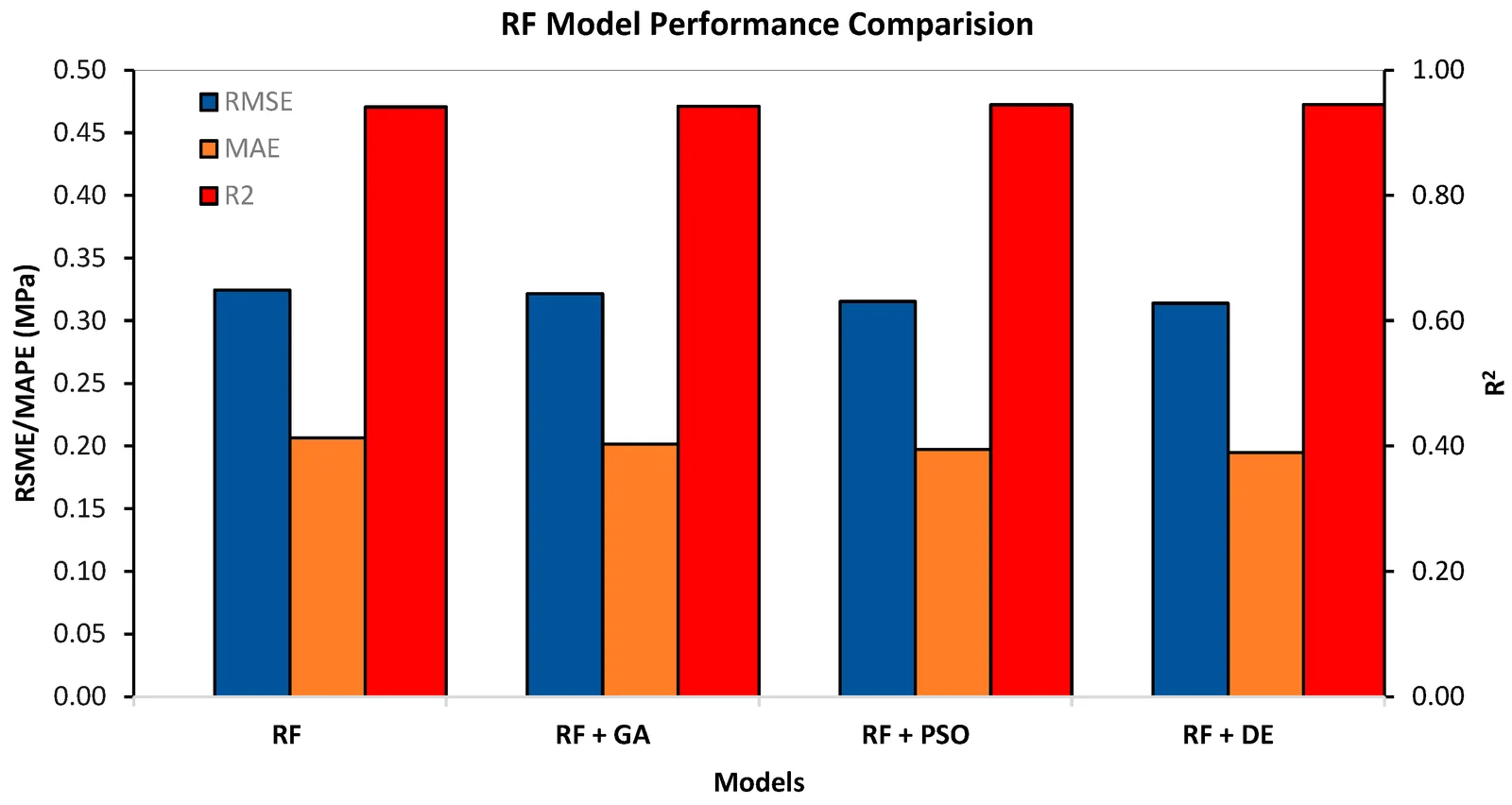
Comparison of error metrics for RF models in STS prediction.

**Figure 8:**
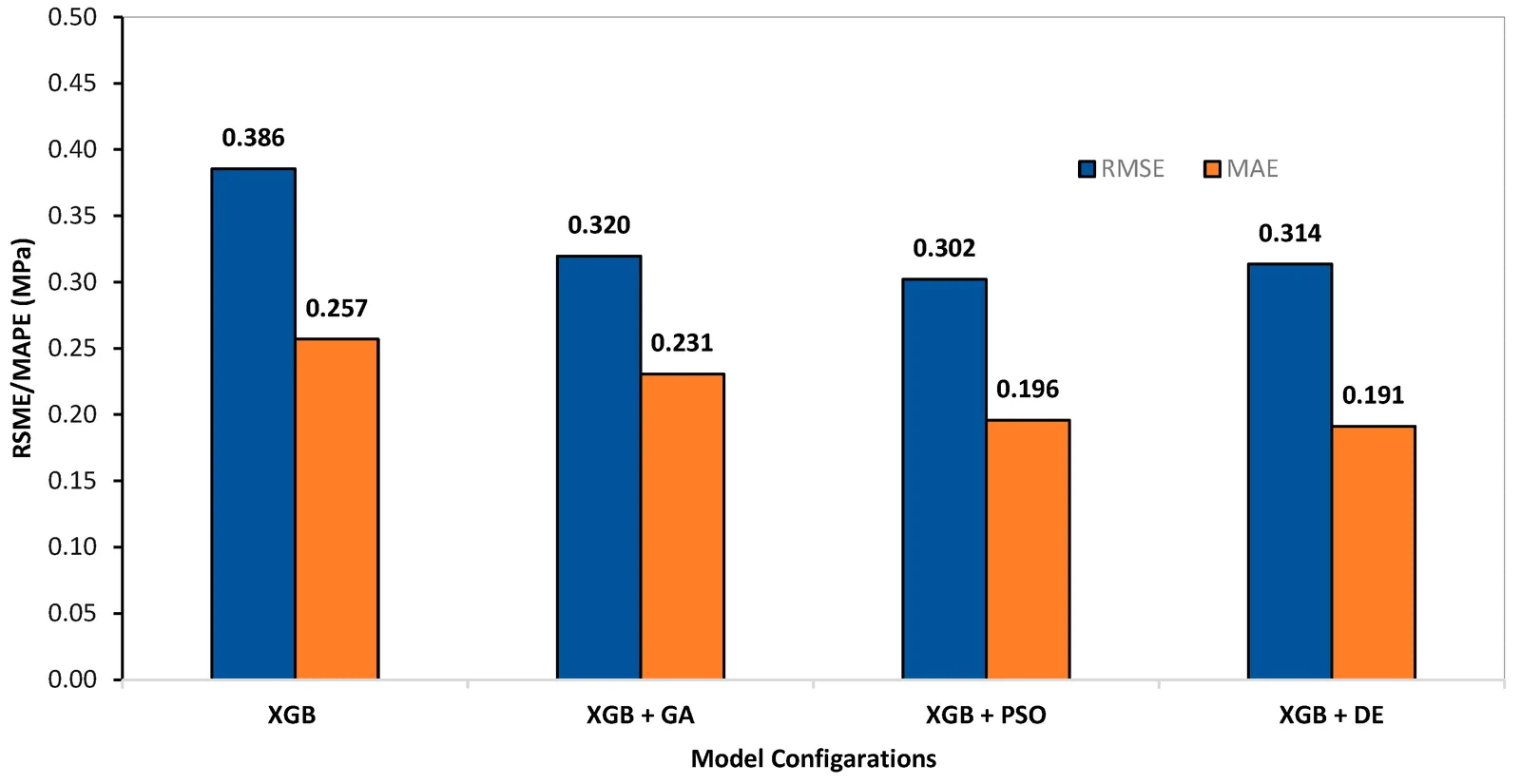
Comparison of error metrics for XGB models in STS prediction.

Figure 6 illustrates that the baseline GBR gives an R^2^ equal to 0.9365 and RMSE equal to 0.338 MPa, and GBR-PSO gives a better result (R^2^ > 0.95, RMSE = 0.289 MPa) at the optimal learning rate, tree depth, and subsampling. Figure 7 shows that RF yields consistent predictions (baseline R^2^ = 0.9413, RMSE = 0.324 MPa), albeit with slight improvement by evolutionary optimization (RF-DE RMSE = 0.314 MPa), but it tends to smooth extreme STS values. As shown in Figure 8, XGBoost, especially XGB-PSO, provides the best predictive accuracy (R^2^ = 0.9491, RMSE = 0.302 MPa), and it is the most effective predictive model for tensile strength, incorporating the nonlinear effects of basalt fibers and outperforms GBR and RF.

**Figure 9:**
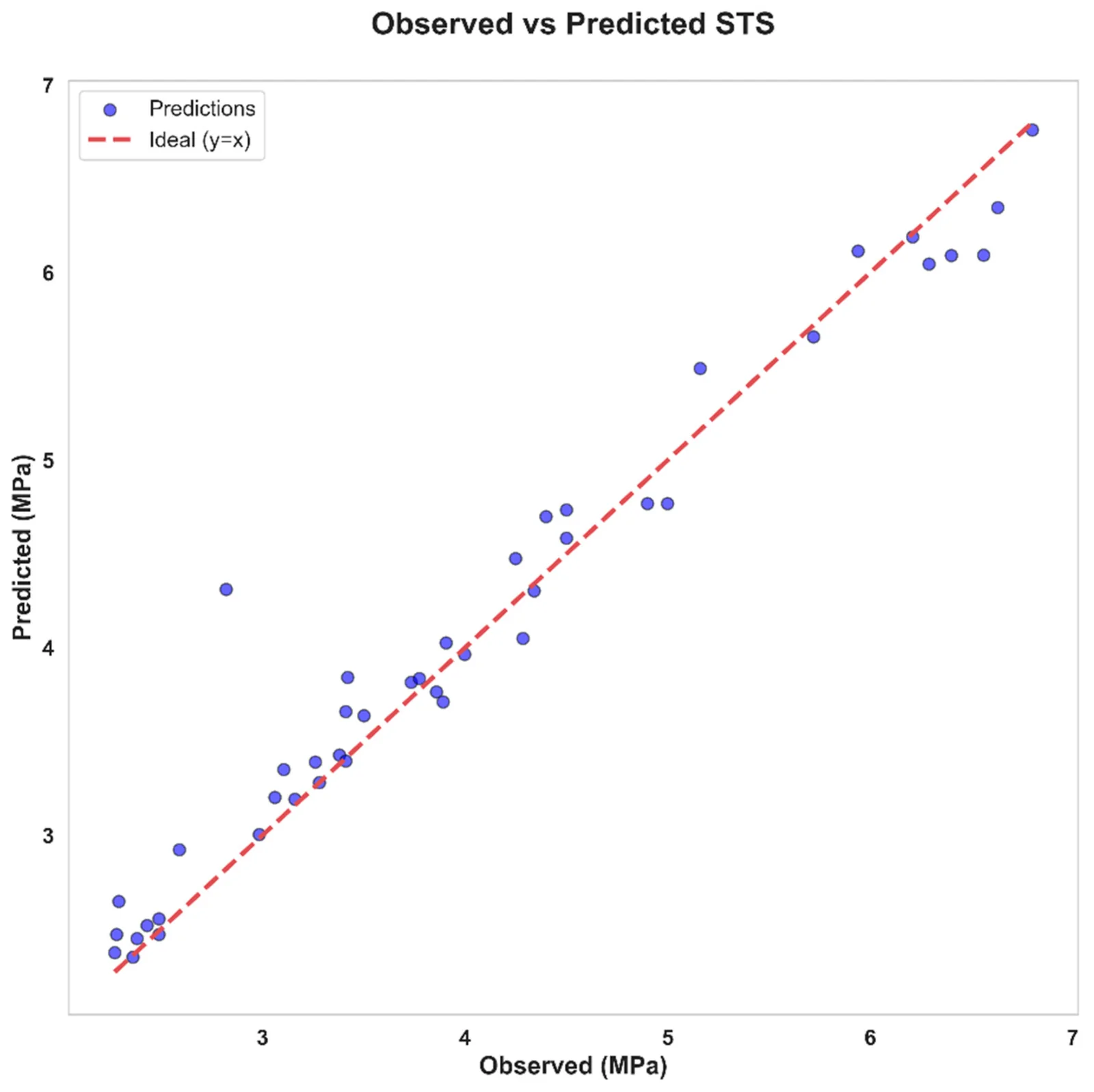
Comparison of observed and predicted STS of BFRC using optimized machine learning models.

Figure 9 illustrates the relationship between the experimentally observed and predicted splitting tensile strength values obtained from the optimized machine learning models. Each data point represents an individual BFRC mix design from the independent test dataset, where the experimentally observed splitting tensile strength is directly paired with the corresponding value predicted by the optimized machine learning model. The data points are closely distributed around the 45° identity line, indicating a high level of agreement between the experimental and predicted values. The slightly lower R^2^ values compared with compressive strength prediction are consistent with the inherent variability of splitting tensile strength measurements, while still demonstrating good prediction accuracy and no evidence of model overfitting.

Table 6 presents three representative BFRC mix designs covering low, medium, and high splitting tensile strength levels from the test dataset. The predicted splitting tensile strength values closely match the corresponding experimental values, with absolute errors below 0.15 MPa and relative errors below 2.5%. These representative cases demonstrate that the optimized machine learning model maintains consistent prediction accuracy across different tensile strength ranges. The results further confirm the robustness and generalization capability of the proposed model for BFRC splitting tensile strength prediction.

### 5.2. Multi-Objective Optimization Results

**Figure 10:**
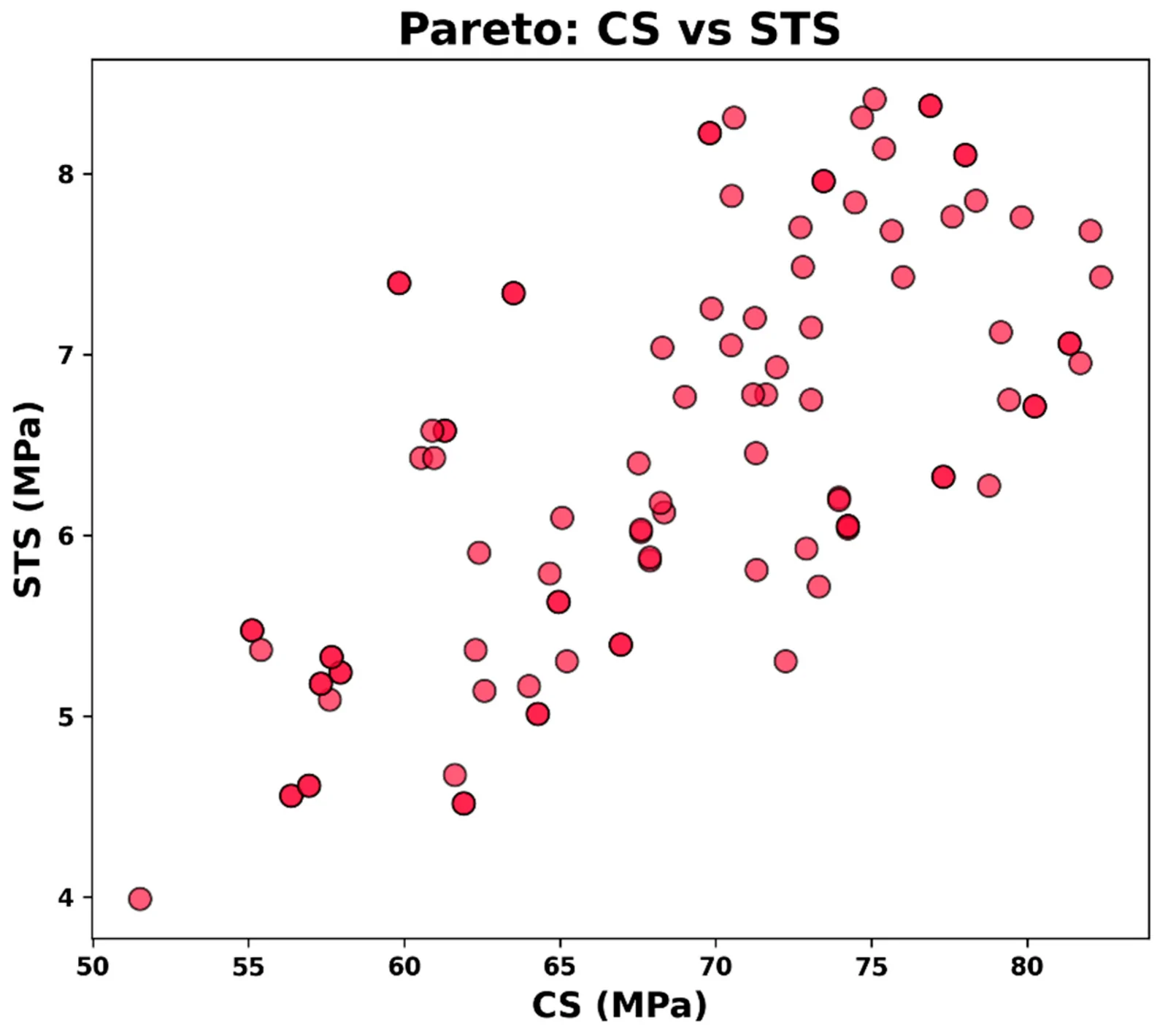
Pareto-optimal front for multi-objective optimization of compressive and splitting tensile strengths of BFRC using NSGA-II.

Figure 10 presents the Pareto-optimal solutions obtained using NSGA-II that optimizes the CS and STS of BFRC at the same time. There is an obvious trade-off: mixes with high CS have moderate STS, but tensile-optimal designs must have a particular fiber structure, which can reduce compressive ability by a few percent. The knee area is the best compromise because it helps to achieve significant tensile gains without significant CS loss. The NSGA-II was effective in searching thousands of mix combinations that give about 50–100 Pareto-optimal solutions that characterize the performance limit.

**Pareto-Optimal Mix Design Characteristics**

Table 7 presents example Pareto-optimal BFRC mixes. Moderate fiber content (~0.08–0.10%) and optimization of cementitious composition result in compressive strength over 80 MPa, and precise fiber length (18–22 mm) and dosage result in splitting tensile strength greater than 8 MPa. The relatively high CS (≈75–80 MPa) and STS (≈8.0–8.4 MPa) are achieved with balanced hybrid designs of the same type, showing the usefulness of multi-objective optimization. Pareto solutions have been classified as minimum-threshold, high-CS, high-STS, and optimal hybrid designs, which can offer a range of performance-based designs for designers based on structural and durability design requirements.

### 5.3. Relative Performance Analysis and Optimization Trends

**Figure 11:**
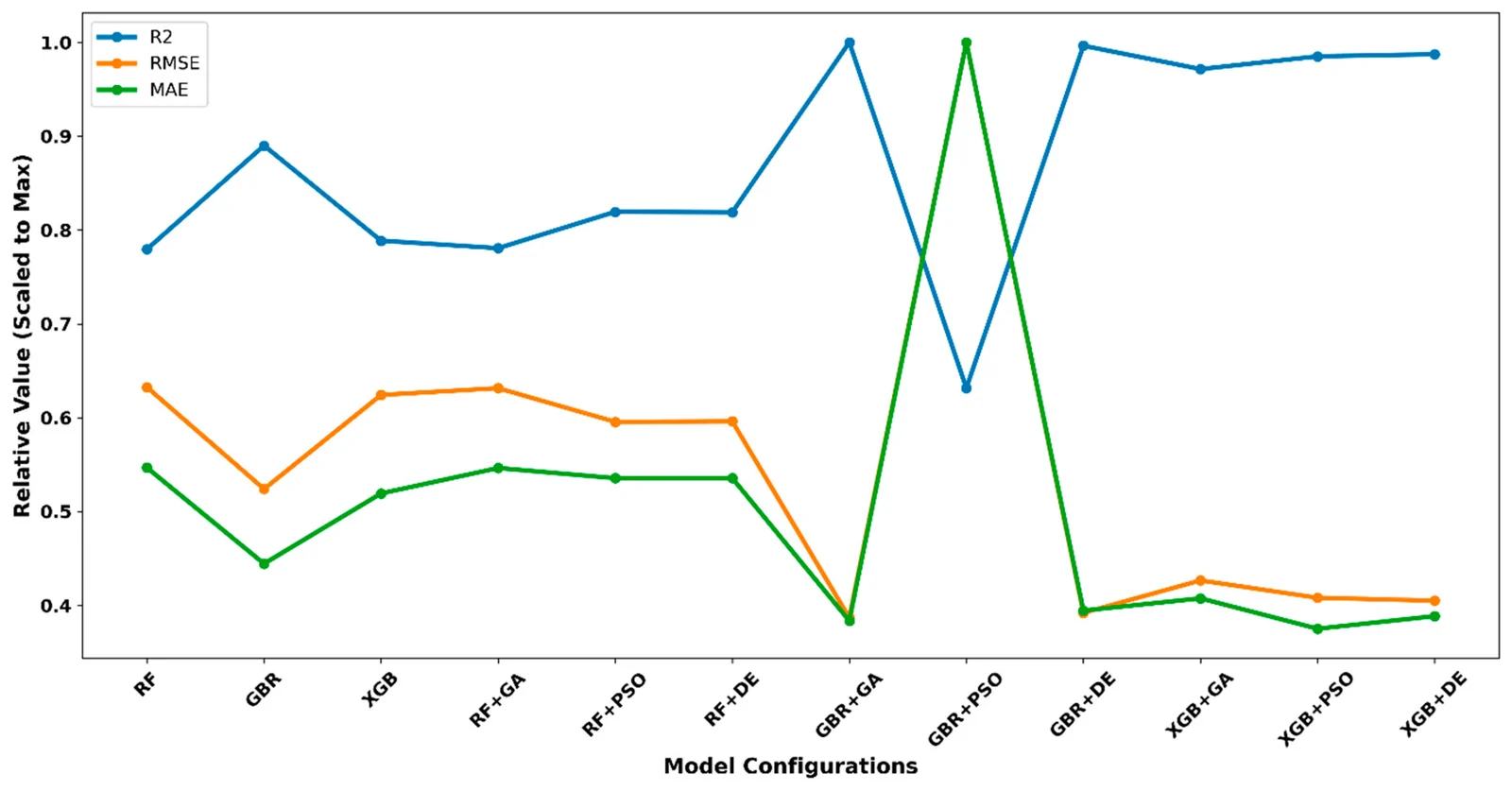
Relative performance measures and optimization patterns for CS prediction with evolutionary-optimized ML models.

**Figure 12:**
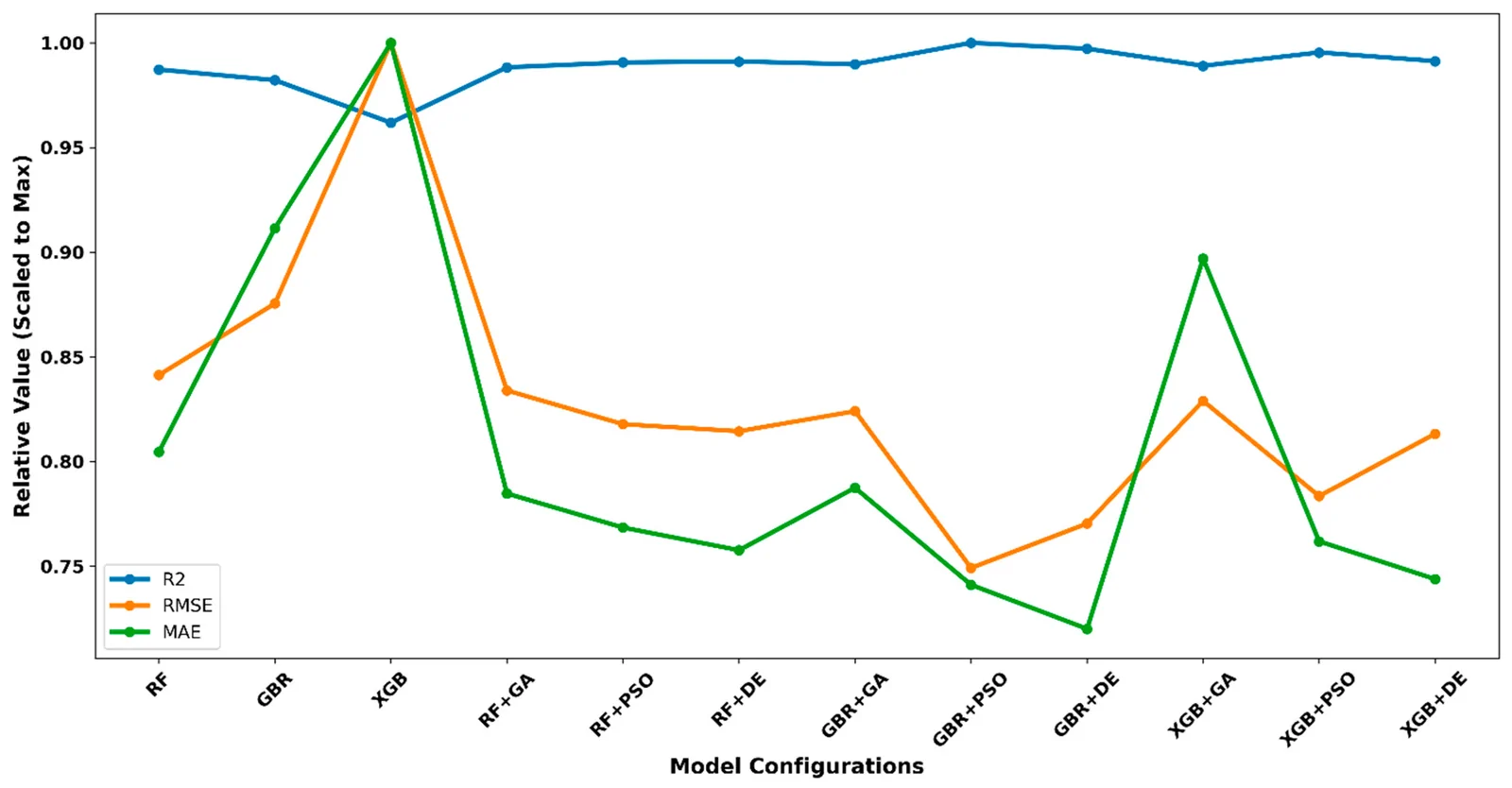
Relative performance of STS prediction with evolutionary-optimized ML models.

Figure 11 and Figure 12 depict the normalized relative performance (R^2^, RMSE, MAE) of CS and STS predictions. In the case of CS, GBR–GA has the most optimal balance of high R^2^ and small errors, whereas GBR-PSO is unstable because of premature convergence. XGBoost is resilient and stable across all optimizers. In the case of STS, GBR-PSO provides the best accuracy, minimal errors, and a smoother learning behavior. In general, the optimization effectiveness depends on the model and target property, which favors the adoption of various evolutionary modeling strategies for performance of BFRC.

### 5.4. Comparative Analysis of Baseline and Optimized Mix Designs

**Figure 13:**
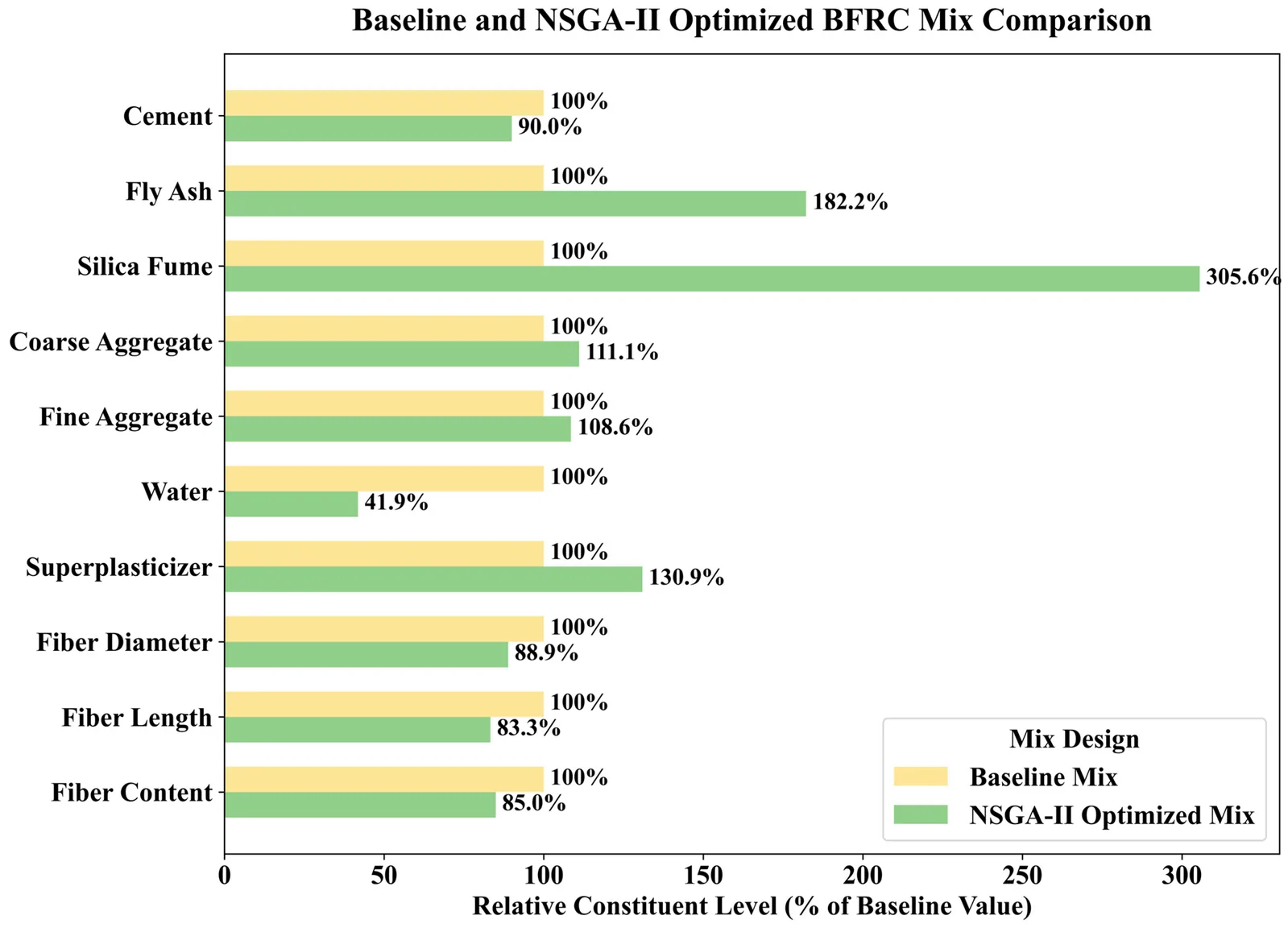
Comparative analysis of material constituents in baseline and NSGA-II–optimized BFRC mix designs.

Figure 13 compares baseline BFRC mixes to NSGA-II-based designs. Optimized mixes show the same or even higher CS and STS due to the redistribution of materials rather than higher utilization. The cement content is decreased by 10–15% through increased use of supplemental materials, the water content is decreased to enhance the densification of the matrix, and the aggregate proportions are changed in order to enhance stress transfer. Basalt fiber content is low (≈0.08–0.12%) and fiber length is slightly shorter, with a greater focus on greater fiber–matrix interaction. In general, the optimized structures improve mechanical efficiency and sustainability.

The feature importance values obtained from SHAP analysis were normalized using Min–Max scaling to transform the original importance scores into a range of 0–1, enabling direct comparison among different input variables. Higher scaled values indicate a greater contribution of the corresponding feature to the model prediction.

### 5.5. Explainable AI Analysis

SHAP analysis was used to interpret the evolutionary machine learning model by quantifying the influence of input variables on CS and STS predictions, revealing feature importance, interactions, and dependency trends.

#### 5.5.1. Global Feature Importance Analysis

**Figure 14:**
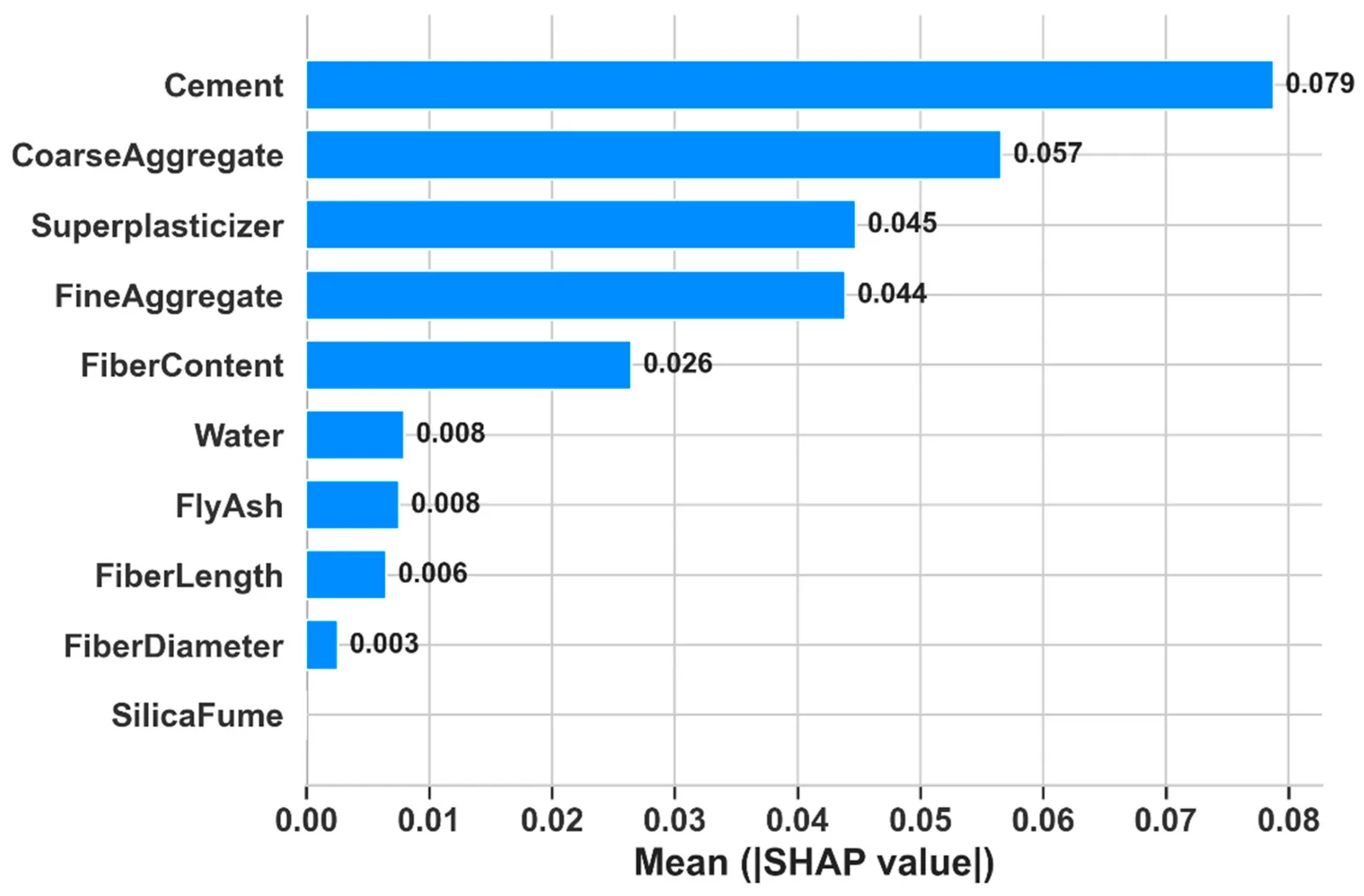
Global SHAP feature importance for CS.

**Figure 15:**
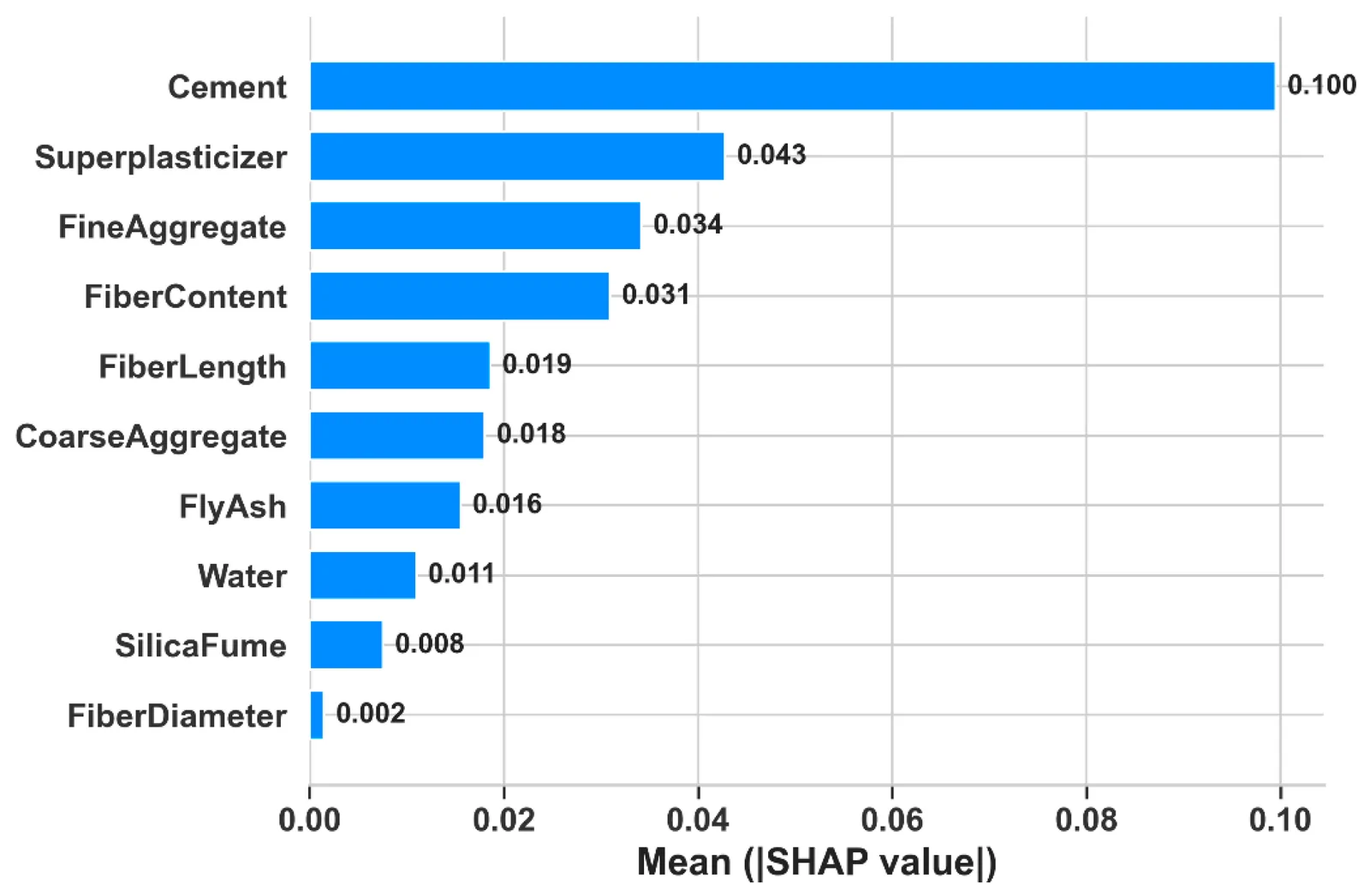
Global SHAP feature importance for STS.

Figure 14 and Figure 15 illustrate SHAP-based feature importance in world features of CS and STS. In the case of CS, the cement content is the most important (≈0.079 MPa), then coarse aggregate, superplasticizer, and fine aggregate, followed by basalt fiber and the rest. In the case of STS, cement is the most pertinent (≈0.100 MPa), superplasticizer and fiber-related characteristics (content and length) become significant, which demonstrates the tensile reinforcing effect of basalt fibers. Aggregates and water are the ones that impact moderately, and the least important are silica fume and fiber diameter.

#### 5.5.2. SHAP Summary Analysis

**Figure 16:**
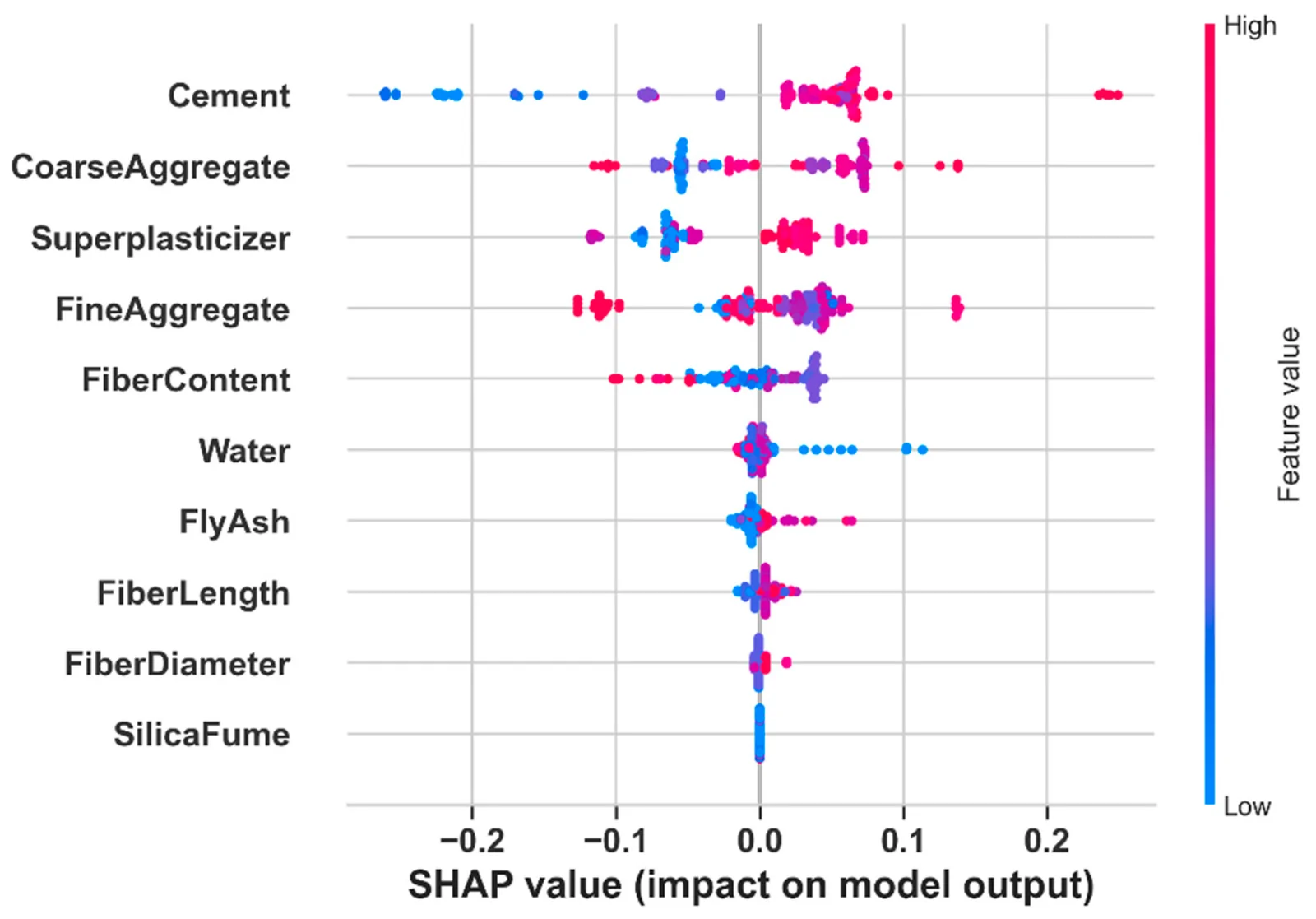
Summary plot for CS prediction.

**Figure 17:**
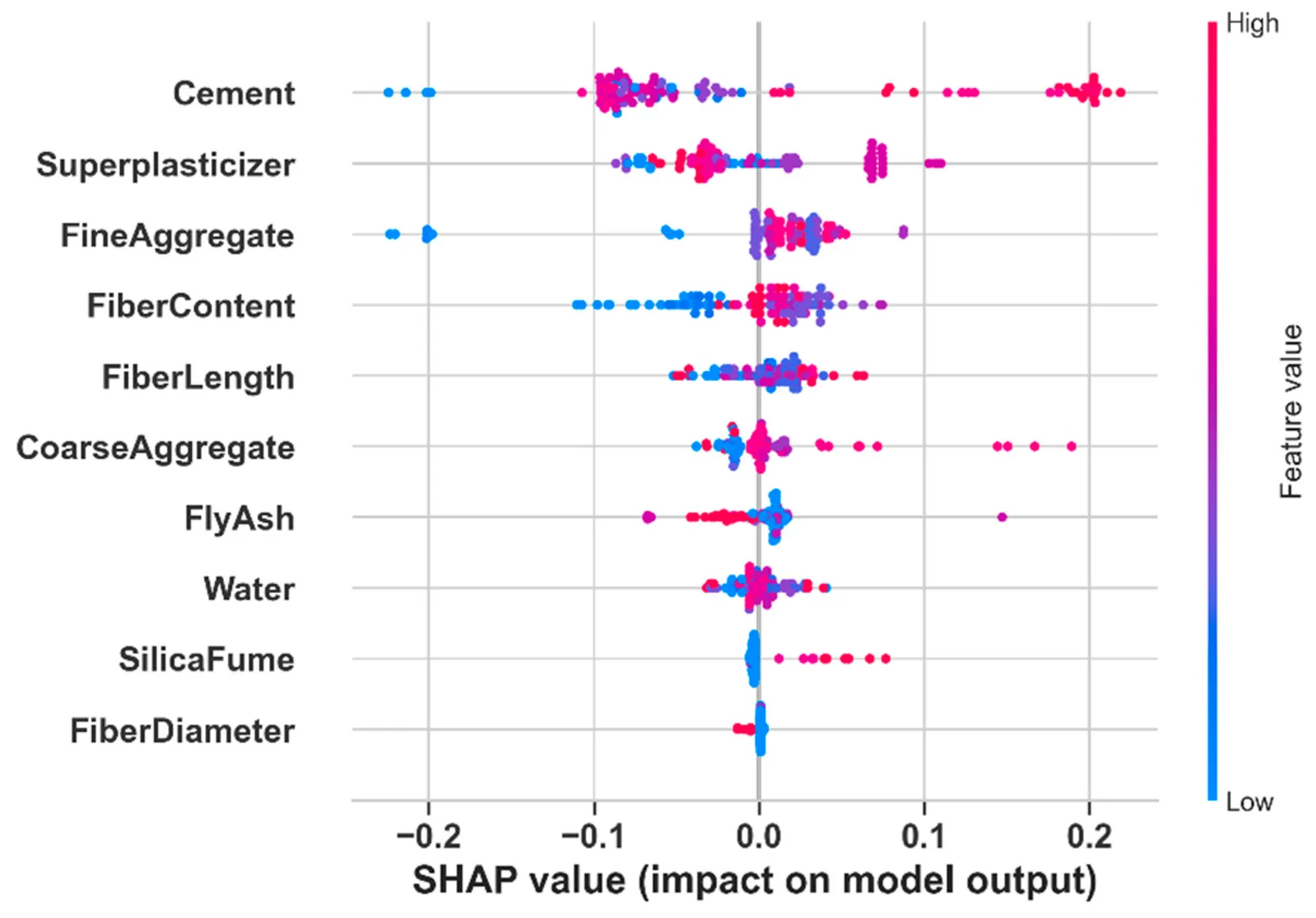
SHAP summary plot for STS prediction.

SHAP summary plots of CS and STS are presented in Figure 16 and Figure 17. In the case of CS, high cement, coarse aggregate, and superplasticizer have a positive effect on predicts whereas low cement or fine aggregate and an excess of fiber may decrease the strength because of porosity. In the case of STS, the content and length of the fiber considerably increase the prediction by crack-bridging, and superplasticizer also has a positive effect, but low fine aggregate has a very negative effect on tensile strength, which is crucial to structural integrity.

#### 5.5.3. SHAP Dependence Analysis

**Figure 18:**
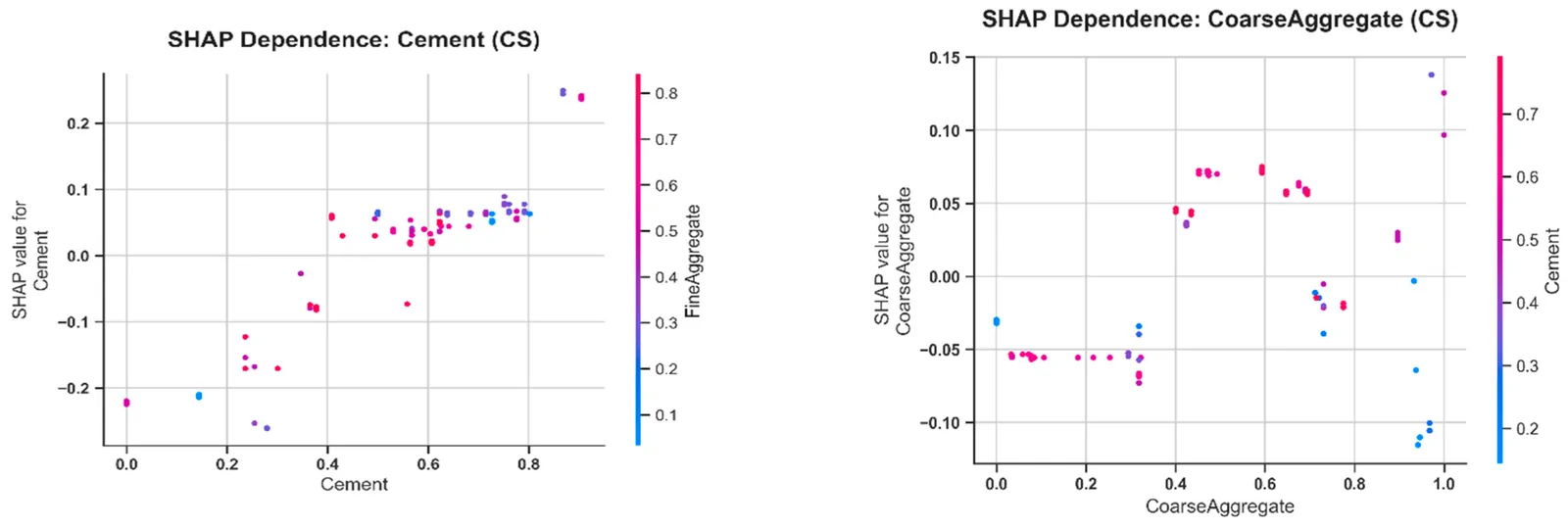

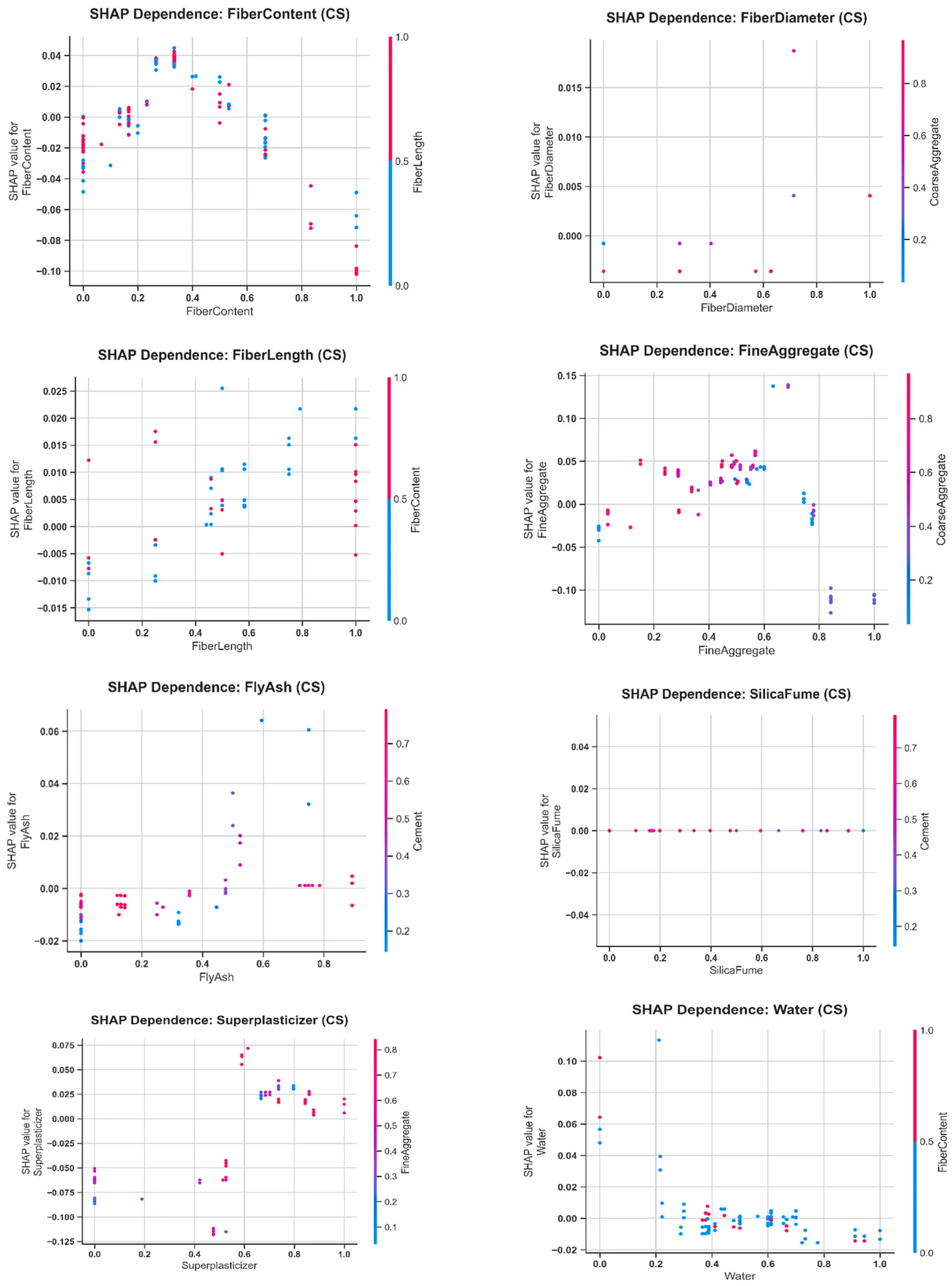
SHAP dependence plot showing feature impact on CS of BFRC.

**Figure 19:**
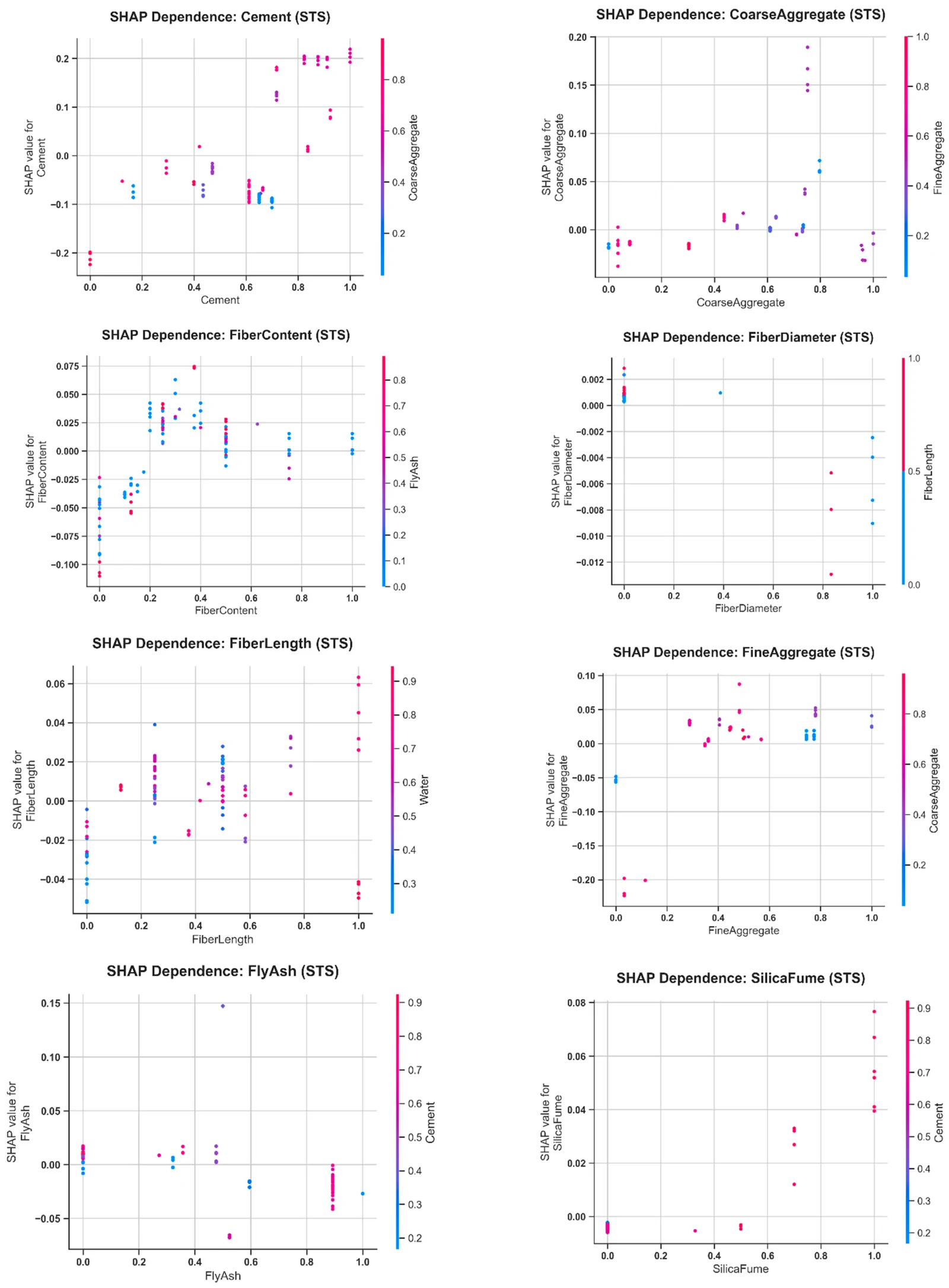

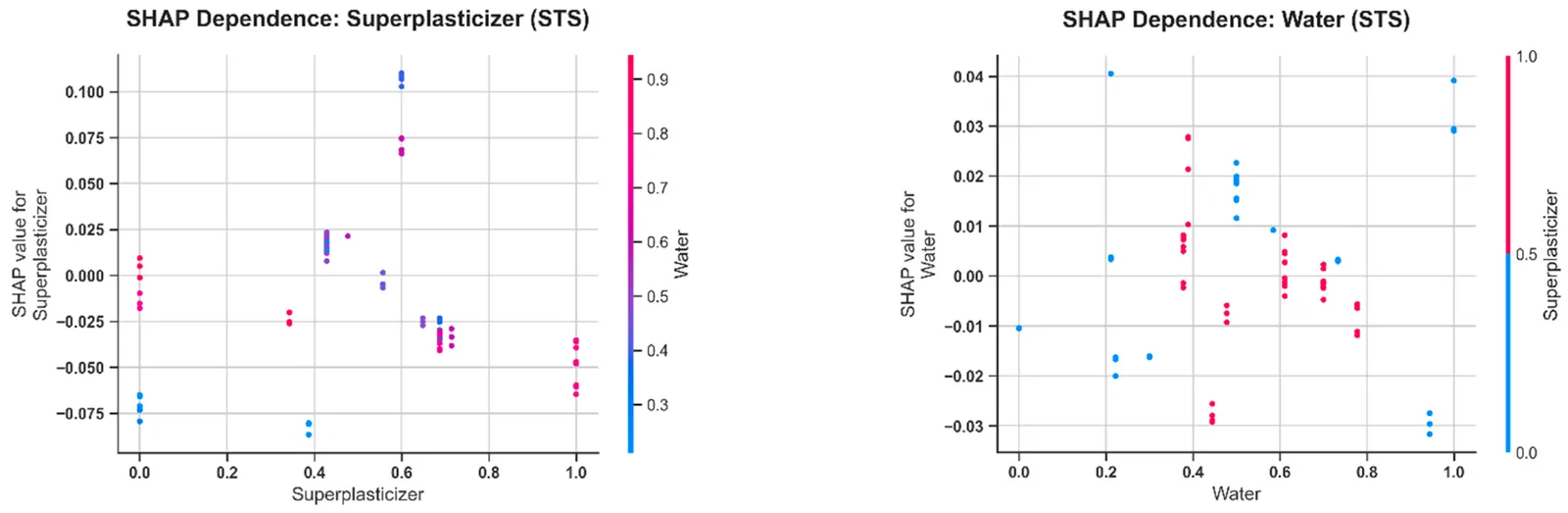
SHAP dependence plot showing feature impact on STS of BFRC.

Figure 18 and Figure 19 give SHAP dependence plots of CS and STS. In the case of CS, cement, silica fume, and superplasticizer have a positive effect on strength, whereas fiber content exhibits a diminishing effect, and fiber length is not very important. In the case of STS, tensile strength is highly promoted by fiber length and smaller diameters; cement affects the performance, and too much fine aggregate or superplasticizer may lower the performance. The content of fiber in food has a weakly decreasing return with high doses, which implies the existence of an optimal level of reinforcement. The SHAP trends are consistent with the mechanical behavior of BFRC. The positive effect of cement content on compressive strength is related to enhanced hydration and matrix densification, while the contribution of basalt fiber content and length to splitting tensile strength is associated with crack bridging and improved stress transfer. Excessive fiber addition may reduce strength due to poor dispersion and increased porosity. These observations demonstrate that the SHAP-based interpretations agree with established concrete material principles.

The SHAP analysis results are also consistent with the established understanding of BFRC mechanical behavior. The higher influence of cement content and superplasticizer dosage can be expected because these parameters directly affect matrix densification, hydration quality, workability, and fiber–matrix bonding. Similarly, the contribution of basalt fiber characteristics, such as fiber length and diameter, is related to crack-bridging capability and stress-transfer efficiency. However, the SHAP analysis provides a quantitative evaluation of the relative importance and nonlinear interactions among these parameters, which cannot be fully identified through conventional experimental observations alone. Therefore, the explainable AI approach complements existing material knowledge by revealing the complex relationships between BFRC mix parameters and mechanical performance.

### 5.6. Graphical User Interface Deployment

**Figure 20:**
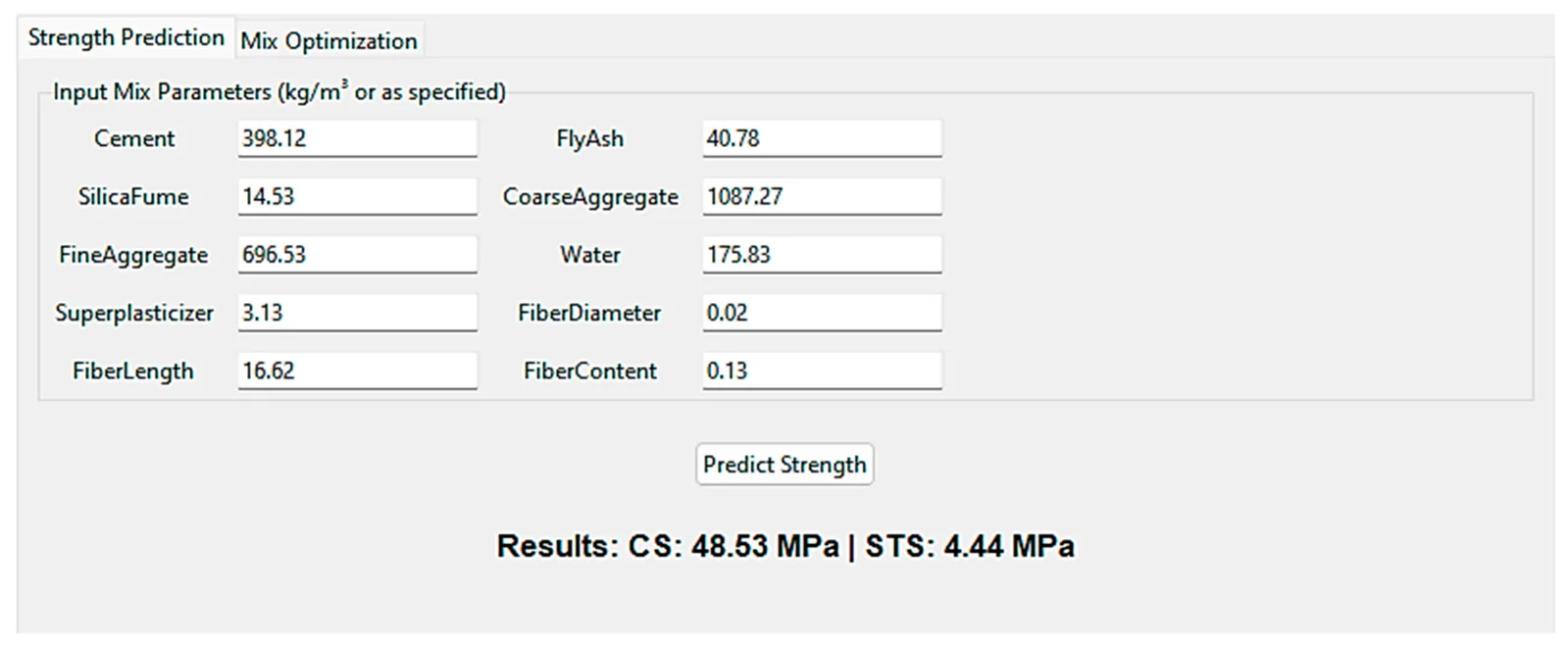
GUI for real-time prediction and optimization of compressive and splitting tensile strengths of BFRC.

The GUI developed to make predictions and optimizations for BFRC strength is shown in Figure 20. Users input material proportions, and the system instantly predicts CS and STS using pre-trained optimized models (GBR–GA for CS and GBR–PSO for STS). In addition to material proportions, the GUI accepts basalt fiber geometrical parameters, including fiber length (6–30 mm) and diameter (0.013–0.020 mm), as input features for strength prediction. These parameters influence crack-bridging efficiency, fiber–matrix interaction, and stress transfer mechanisms, thereby affecting both compressive and splitting tensile strength predictions. For the sample mix (cement 398.12 kg/m^3^, water 175.83 kg/m^3^, basalt fiber content 0.13%), the GUI predicts CS = 48.53 MPa and STS = 4.44 MPa, demonstrating reliable real-time performance. The interface also provides access to Pareto-optimal mix recommendations, supporting efficient strength–sustainability trade-off decisions for civil engineers.

## 6. Discussion

The research demonstrates that the evolutionary-optimized ensemble machine learning models are highly effective when it comes to the prediction of the mechanical performance of BFRC. The analyzed models, GBR optimized by GA and PSO, showed the highest accuracy for compressive and splitting tensile strength, respectively, indicating the necessity of optimizer–model compatibility with particular mechanical responses. The variation in prediction performance highlights the importance of optimizer–model compatibility. GA provides effective global exploration for GBR hyperparameter tuning, while PSO achieves efficient convergence for XGBoost through balanced exploration and exploitation. DE offers stable optimization through population diversity. These results indicate that selecting an appropriate evolutionary algorithm according to the ML model characteristics is essential for achieving optimal performance. The AI analysis using SHAP showed cement content to be the biggest influence on both strength measures, with the basalt fiber parameters being the second most influential on compression strength and the first influence on tensile behavior. The improvement of the fiber content and length in the STS was found to be numerically justified by the crack-bridging effect of basalt fibers, but excessively high doses of fiber were observed to adversely affect the compressive strength due to the high porosity. The multi-objective optimization model worked well to identify the Pareto-optimal mix designs to achieve strength improvement with low cement consumption, supporting sustainable concrete production. These optimized designs support green concrete principles by achieving improved mechanical performance with reduced material demand, thereby contributing to lower environmental impact associated with concrete production. Additionally, the suggested framework will be transformed into a feasible engineering tool, enabling real-time strength prediction and optimal mix choice through the implementation of a GUI. The SHAP-based explainable AI improves the transparency of the optimized prediction framework by identifying the contribution of individual mix parameters. While SHAP has previously been applied for interpreting BFRC performance, the proposed framework integrates it with evolutionary optimization and GUI-based decision support to enhance practical engineering applications. Compared with previous studies that combined machine learning with NSGA-II for concrete optimization, the proposed framework extends the existing literature by integrating evolutionary hyperparameter optimization (GA, PSO, and DE), SHAP-based explainable artificial intelligence for model interpretation, and GUI-based deployment for real-time engineering decision support. These additional components enhance model transparency, optimization flexibility, and engineering applicability while maintaining accurate prediction and optimization performance.

Despite the effectiveness of the proposed framework, some limitations should be acknowledged. The dataset is mainly based on laboratory-derived BFRC mixtures and may not fully capture field variations in material sources, curing conditions, and construction practices. Additionally, the dataset size may affect model generalization; therefore, further validation with larger and more diverse datasets is required to improve robustness and applicability. Another limitation of this study is that the dataset is restricted to 28-day strength measurements. Although 28-day strength is a widely adopted benchmark for concrete evaluation, variations in curing age and environmental exposure conditions can influence the long-term mechanical performance of BFRC. Incorporating additional parameters, such as different curing periods and durability-related factors, could further enhance the generalizability and applicability of the proposed framework.

## 7. Conclusions

- This research demonstrates the efficiency of the evolutionary ML methods to forecast the mechanical behavior of BFRC accurately.
- The results of the experiment show that ensemble- and boosting-based models, which are optimized with evolutionary algorithms, are more efficient than traditional machine learning methods.
- The highest-performing models achieved R^2^ = 0.88 for CS and 0.95 for STS, with low prediction errors (RMSE = 2.80 MPa and 0.19 MPa) in modeling material and nonlinear behavior.
- Model convergence, model stability, and model generalization among a wide range of BFRC mix compositions were improved when GA, PSO, and DE were used to tune the hyperparameters.
- Stable results in various statistical measures and explainability using SHAP confirmed the reliability of the model, distinguished the key input parameters, and ensured interpretability, which is essential in engineering practice.
- The proposed framework offers an effective decision-support tool for the industrial design of BFRC mixes, enabling rapid strength estimation, reducing experimental resource consumption, minimizing material waste, and improving overall quality control.
- It is also observed that the evolutionary machine learning system offers a scalable, accurate, and realistic method for performance-based and sustainable construction, as well as data-driven material design in current civil engineering.

### Future Scope

Future studies can focus on expanding the dataset with additional experimental results, incorporating other mechanical properties such as shear strength, and validating the proposed framework using large-scale field applications. The integration of advanced hybrid learning techniques and real-time monitoring data can further enhance the practical applicability of BFRC performance prediction and sustainable mix design optimization.

## Figures and Tables

**Table 1:** Description of the input and output variables used in the BFRC dataset.

Variable	Symbol	Type	Description	Unit
Cement content	C	Input	Amount of cement in the mix	kg/m^3^
Water content	W	Input	Water used in the concrete mix	kg/m^3^
Fine aggregate	FA	Input	Fine aggregate content	kg/m^3^
Coarse aggregate	CA	Input	Coarse aggregate content	kg/m^3^
Water–cement ratio	W/C	Input	Ratio of water to cement	–
Superplasticizer	SP	Input	Superplasticizer dosage	kg/m^3^
Basalt fiber content	BF	Input	Fiber dosage	% or kg/m^3^
Fiber length	FL	Input	Length of basalt fibers	mm
Fiber diameter (or aspect ratio, if applicable)	FD	Input	Fiber geometry parameter	mm
Curing age	Age	Input	Age of specimen	days
Compressive strength	CS	Output	Compressive strength	MPa
Splitting tensile strength	STS	Output	Splitting tensile strength	MPa
Sample ID (if included)	ID	Dataset	Sample identifier	–

**Table 2:** Hyperparameter configuration and optimal values for evolutionary-optimized ML models.

Algorithm	Parameter	Candidate Values	Selected (CS)	Selected (STS)
**Random Forest**	Number of Trees	50–300	80	100
	Tree Depth	3–20	16	11
	Feature Subset Size	2–12	4	5
	Minimum Split Samples	2–10	2	2
	Minimum Leaf Samples	1–4	1	2
**Gradient Boosting**	Number of Boosting Stages	50–500	70	100
	Learning Rate	0.01–0.2	0.2	0.05
	Maximum Depth	3–11	5	5
	Subsample Ratio	0.1–1.0	0.8	1.0
**XGBoost**	Number of Trees	50–500	100	200
	Learning Rate	0.01–0.2	0.1	0.05
	Tree Depth	3–11	5	7
	Row Sampling Ratio	0.5–1.0	0.8	1.0
	Feature Sampling Ratio	0.5–1.0	0.7	0.8

**Table 3:** Performance evaluation metrics of baseline and evolutionary-optimized machine learning models for CS prediction.

Model Configuration	R^2^	RMSE (MPa)	MAE (MPa)
RF (Baseline)	0.6888	4.5640	2.9915
GBR (Baseline)	0.7864	3.7814	2.4319
XGB (Baseline)	0.6968	4.5048	2.8416
RF + GA	0.6898	4.5569	2.9897
RF + PSO	0.7241	4.2979	2.9304
RF + DE	0.7236	4.3015	2.9310
GBR + GA	0.8836	2.7914	2.0993
GBR + PSO	0.5581	7.2160	5.4718
GBR + DE	0.8804	2.8291	2.1583

**Table 4:** Representative BFRC mix design prediction cases.

Sample	Strength Level	Observed CS (MPa)	Predicted CS (MPa)	Absolute Error (MPa)	Relative Error (%)
Sample 1	Low	28.60	29.12	0.52	1.82
Sample 2	Medium	54.80	55.34	0.54	0.99
Sample 3	High	82.40	81.73	0.67	0.81

**Table 5:** Performance evaluation metrics of baseline and evolutionary-optimized machine learning models for STS prediction.

Model Configuration	R^2^	RMSE (MPa)	MAE (MPa)
RF (Baseline)	0.9413	0.3244	0.2067
GBR (Baseline)	0.9365	0.3375	0.2342
XGB (Baseline)	0.9171	0.3855	0.2569
RF + GA	0.9424	0.3215	0.2016
RF + PSO	0.9446	0.3153	0.1974
RF + DE	0.9450	0.3140	0.1947
GBR + GA	0.9437	0.3177	0.2023
GBR + PSO	0.9535	0.2888	0.1904
GBR + DE	0.9508	0.2970	0.1850
XGB + GA	0.9431	0.3196	0.2305
XGB + PSO	0.9491	0.3021	0.1958
XGB + DE	0.9452	0.3135	0.1911

**Table 6:** Representative BFRC mix design prediction cases.

Sample	Strength Level	Observed STS (MPa)	Predicted STS (MPa)	Absolute Error (MPa)	Relative Error (%)
Sample 1	Low	**1.74**	**1.78**	**0.04**	**2.30**
Sample 2	Medium	**3.72**	**3.75**	**0.03**	**0.81**
Sample 3	High	**9.80**	**9.67**	**0.13**	**1.33**

**Table 7:** NSGA-II optimization Pareto-optimal mix designs.

Solution Type	Cement (kg/m^3^)	Water (kg/m^3^)	Fiber Content (%)	Fiber Length (mm)	CS (MPa)	STS (MPa)
**Minimum-Threshold Design**	380	170	0.05	12	62.4	5.6
**High-CS Design**	440	160	0.08	16	82.3	7.1
**High-CS Design**	430	165	0.10	18	80.7	7.4
**High-STS Design**	400	175	0.15	22	71.6	8.6
**High-STS Design**	390	178	0.14	20	69.8	8.3
**Optimal Hybrid Design (Knee Point)**	415	168	0.10	18	78.4	8.2
**Optimal Hybrid Design**	420	170	0.09	16	75.9	8.0

## Data Availability

The original contributions presented in this study are included in the article. Further inquiries can be directed to the corresponding authors.

## Abbreviations

**Abbreviation**	**Full Form**
BFRC	Basalt fiber-reinforced concrete
CS	Compressive strength
DE	Differential evolution
DEAP	Differential Evolution with Adaptive Parameters
GBR	Gradient Boosting Regressor
GA	Genetic Algorithm
GUI	Graphical User Interface
MAE	Mean Absolute Error
ML	Machine Learning
NSGA-II	Non-dominated Sorting Genetic Algorithm II
PSO	Particle Swarm Optimization
R^2^	Coefficient of Determination
RF	Random Forest
RMSE	Root Mean Square Error
SHAP	SHapley Additive exPlanations
STS	Splitting tensile strength
XAI	Explainable artificial intelligence
XGBoost	eXtreme Gradient Boosting
